# Transcriptional Profiling of Cultured, Embryonic Epicardial Cells Identifies Novel Genes and Signaling Pathways Regulated by TGFβR3 *In Vitro*

**DOI:** 10.1371/journal.pone.0159710

**Published:** 2016-08-09

**Authors:** Daniel M. DeLaughter, Cynthia R. Clark, Danos C. Christodoulou, Christine E. Seidman, H. Scott Baldwin, J. G. Seidman, Joey V. Barnett

**Affiliations:** 1 Department of Cell and Developmental Biology, Vanderbilt University School of Medicine, Nashville, Tennessee, United States of America; 2 Department of Pharmacology, Vanderbilt University School of Medicine, Nashville, Tennessee, United States of America; 3 Department of Genetics, Harvard Medical School, Boston, Massachusetts, United States of America; 4 Cardiology Division, Brigham and Women's Hospital and Harvard Medical School, Boston, Massachusetts, United States of America; 5 Department of Pediatrics, Vanderbilt University School of Medicine, Nashville,Tennessee, United States of America; IRCCS San Raffaele Pisana, ITALY

## Abstract

The epicardium plays an important role in coronary vessel formation and *Tgfbr3*^-/-^ mice exhibit failed coronary vessel development associated with decreased epicardial cell invasion. Immortalized *Tgfbr3*^-/-^ epicardial cells display the same defects. *Tgfbr3*^+/+^ and *Tgfbr3*^-/-^ cells incubated for 72 hours with VEH or ligands known to promote invasion via TGFβR3 (TGFβ1, TGFβ2, BMP2), for 72 hours were harvested for RNA-seq analysis. We selected for genes >2-fold differentially expressed between *Tgfbr3*^+/+^ and *Tgfbr3*^-/-^ cells when incubated with VEH (604), TGFβ1 (515), TGFβ2 (553), or BMP2 (632). Gene Ontology (GO) analysis of these genes identified dysregulated biological processes consistent with the defects observed in *Tgfbr3*^-/-^ cells, including those associated with extracellular matrix interaction. GO and Gene Regulatory Network (GRN) analysis identified distinct expression profiles between TGFβ1-TGFβ2 and VEH-BMP2 incubated cells, consistent with the differential response of epicardial cells to these ligands *in vitro*. Despite the differences observed between *Tgfbr3*^+/+^ and *Tgfbr3*^-/-^ cells after TGFβ and BMP ligand addition, GRNs constructed from these gene lists identified NF-ĸB as a key nodal point for all ligands examined. *Tgfbr3*^-/-^ cells exhibited decreased expression of genes known to be activated by NF-ĸB signaling. NF-ĸB activity was stimulated in *Tgfbr3*^+/+^ epicardial cells after TGFβ2 or BMP2 incubation, while *Tgfbr3*^-/-^ cells failed to activate NF-ĸB in response to these ligands. *Tgfbr3*^+/+^ epicardial cells incubated with an inhibitor of NF-ĸB signaling no longer invaded into a collagen gel in response to TGFβ2 or BMP2. These data suggest that NF-ĸB signaling is dysregulated in *Tgfbr3*^-/-^ epicardial cells and that NF-ĸB signaling is required for epicardial cell invasion *in vitro*. Our approach successfully identified a signaling pathway important in epicardial cell behavior downstream of TGFβR3. Overall, the genes and signaling pathways identified through our analysis yield the first comprehensive list of candidate genes whose expression is dependent on TGFβR3 signaling.

## Introduction

The epicardium plays an important role in coronary vessel development (reviewed [1–3]). Formation of the epicardium occurs when a population of mesothelial cells, termed the proepicardium, attach to and migrate over the heart tube myocardium [4, 5]. Subsequently, a subpopulation of the epithelial, epicardial cells lose epithelial character, change shape, and invade the underlying matrix in a process termed epithelial-mesenchymal transformation (EMT). The resulting mesenchymal cells invade into the subepicardial space with some cells proceeding to invade into the myocardium as well (reviewed [6]). These epicardial-derived cells differentiate into distinct lineages [7–11], that include cardiac fibroblasts, pericytes, and vascular smooth muscle cells, and support the formation of coronary vessels. Several reports support epicardial contribution to the coronary endothelial cell lineage [12–14]. Numerous lines of evidence are now revealing the importance of these same developmental processes in cardiac repair and that the epicardium makes critical contributions to cardiac response to injury (reviewed [6]). Despite this, the signaling processes which regulate epicardial EMT are incompletely understood.

TGFβR3 deletion in mice leads to failed coronary vessel development [15]. *Tgfbr3*^*-/-*^ hearts featured a discontinuous epicardium overlying an expanded subepicaridal space. Further studies revealed a significant decrease in proliferation and invasion of epicardium and epicardially-derived mesenchyme [16]. Overall, these studies demonstrated that TGFβR3 plays an important and non-redundant role in epicardial behavior and coronary vessel development *in vivo*.

TGFβR3 binds TGFβ1, TGFβ2 and TGFβ3 and is uniquely required to bind TGFβ2 with high affinity [17, 18]. TGFβR3 is also capable of binding and signaling in response to BMP2 [19] and functions as a receptor for inhibin [20]. TGFβR3 presents ligand to TGFβR2 to promote both Smad-dependent and -independent signaling [21]. The highly conserved 43 amino acid intracellular domain of TGFβR3 is not required for ligand presentation [22] but may regulate other signaling events. Phosphorylation of the cytoplasmic domain of TGFβR3 by TGFβR2 at Thr841 is required for β-arrestin2 binding, leading to internalization of TGFβR3 and down-regulation of TGFβ signaling. The 3 C-terminal amino acids of TGFβR3, STA, are a Class I PDZ binding domain that binds the scaffolding protein GIPC which in turn stabilizes TGFβR3 on the plasma membrane to promote signaling [23].

*Tgfbr3*^*+/+*^ epicardial cells undergo loss of epithelial character and invasion into collagen gels *in vitro* in response to TGFβ1, TGFβ2, and BMP2, ligands known to bind TGFβR3 [18, 24]. While loss of epithelial character was still observed after loss of TGFβR3, *Tgfbr3*^*-/-*^ cells had reduced invasion in response TGFβ1, TGFβ2, and BMP2, a response that was rescued by the addition of TGFβR3 [16, 25, 26]. TGFβ1 and TGFβ2 promoted smooth muscle differentiation in *Tgfbr3*^*+/+*^ and *Tgfbr3*^*-/-*^ cells while BMP2 did not [26]. Surprisingly, other ligands known to be important in epicardial EMT also required TGFβR3 to promote invasion in epicardial cells (FGF2 [27, 28], High Molecular Weight HMW-HA [29, 30]). Impaired invasion of three-dimensional gels by epicardial-derived mesenchyme was not due to the permanent loss of invasive properties, as PDGFAA, PDGFBB and VEGFC still induced invasion in *Tgfbr3*^*-/-*^ epicardial cells [16].

This ability of TGFβR3 to regulate epicardial cell behavior in response to an array of ligands may explain the severity of the *in vivo* phenotype of *Tgfbr3*^*-/-*^ embryos when compared to the absence of a phenotype in mice lacking individual TGFβ ligands [31–33]. It is known that the loss of cell invasion has effects on cardiovascular development outside of the loss of the direct contributions of these cells to the structure of the coronary vessels. The deletion of several genes, encoding proteins that perform an array of functions including transcription factors, adhesion molecules, and growth factor ligands or receptors, share a common phenotype of a thinned myocardium (reviewed in [3]). These data as well as experimental embryology experiments in avian embryos have been interpreted to demonstrate that epicardially-derived mesenchymal cells are necessary for growth of the compact zone of the myocardium (reviewed in [34]). Therefore, the formation of the epicardium and the resultant generation of mesenchyme is critical for the support of both coronary vessel formation and myocardial growth. For example, targeted deletion of ALK5 in the epicardium in mice *in vivo* results in interrupted epicardial attachment to the myocardium, loss of expression of specific adhesion molecules, thinned myocardium, and a loss of coronary smooth muscle [33]. These embryos survive until birth, suggesting that, unlike in embryos lacking TGFβR3, the coronary vessels function to some degree as mice lacking coronary vessels die at approximately E14.5-E16.5 [35–37]. These data suggest that TGFβR3 signaling regulates a common pathway accessed by several upstream regulators of cell invasion.

TGFβR3-dependent invasion stimulated by TGFβ1, TGFβ2, BMP2, HMW-HA, or FGF2 was shown to require the cytoplasmic domain of TGFβR3 *in vitro* [16]. Overexpression of TGFβR3 rescued invasion in *Tgfbr3*^*-/-*^ epicardial cells *in vitro* in response to TGFβ1, TGFβ2, BMP2, HMW-HA, or FGF2, whereas constructs expressing a TGFβR3 mutant lacking the 3 C-terminal amino acids required for GIPC binding fail to rescue invasion [16, 25, 26]. The importance of this interaction is further supported by the observation that GIPC is not only required for invasion in *Tgfbr3*^*+/+*^ epicardial cells, but GIPC overexpression can promote invasion in the absence of additional ligand. GIPC regulation of epicardial invasion depends on TGFβR3 since GIPC expression in *Tgfbr3*^*-/-*^ cells fails to rescue invasion and inhibition of GIPC expression impairs the ability of TGFβR3 to rescue invasion in *Tgfbr3*^*-/-*^ cells [16]. Similar results were observed in endocardial cushions where the interaction of TGFβR3 with GIPC is required to promote TGFβ2- and BMP2- dependent invasion *in vitro* [38]. These data linking defects in invasion of *Tgfbr3*^*-/-*^ epicardial cells to the cytoplasmic domain of TGFβR3, which is not required for ligand presentation, suggests a unique, non-redundant role for TGFβR3 in regulating epicardial and endocardial EMT.

Here, we use a well defined *in vitro* system based on immortalized epicardial cells coupled with RNA-seq analysis to generate a transcriptional profile of *Tgfbr3*^*+/+*^ *and Tgfbr3*^*-/-*^ cells incubated with ligands that stimulate TGFβR3-dependent invasion. The resulting transcriptional profiles have identified regulators of epicardial cell behavior downstream of TGFβR3 and provided the first description of genes downstream of TGFβR3.

## Methods

### Generation of cell lines

**Fig 1:**
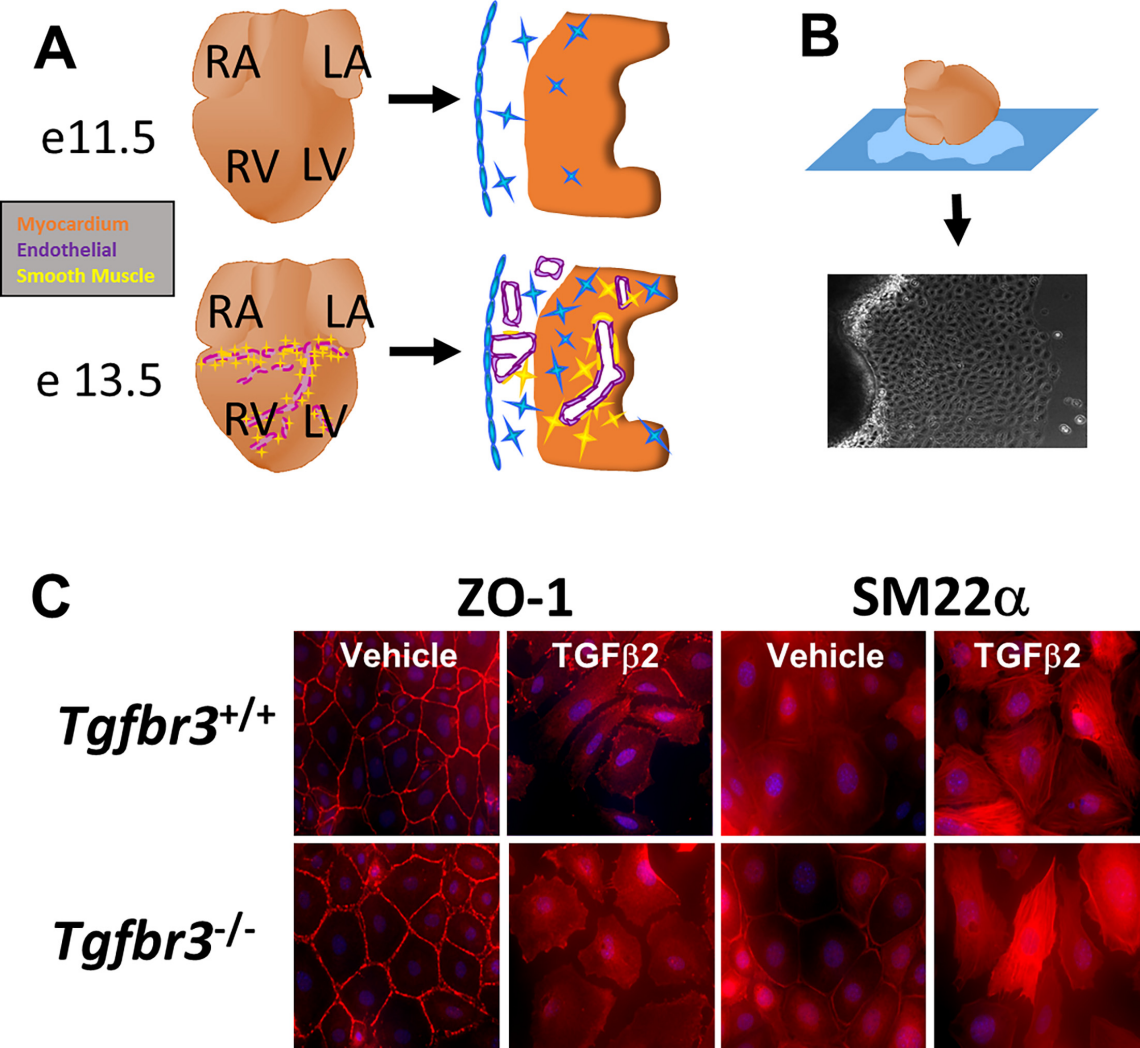
Immortalized epicardial cells undergo loss of epithelial character. (A) The epicardium undergoes EMT at E11.5–13.5. Subsequently, transforming epicardial cells invade the subepicardial space and myocardium towards forming coronary vessels. Blue- epicardium. Purple- endothelium. Yellow- smooth muscle cells. Red- myocardium. (B) Immortalized epicardial cells were derived from E11.5 *Tgfbr3*^*+/+*^ and *Tgfbr3*^-/-^ embryos which expressed a temperature-sensitive large T-antigen. (C) Immunohistochemistry of *Tgfbr3*^*+/+*^ or *Tgfbr3*^-/-^ immortalized epicardial cells after 72 hours incubation with TGFβ2 or vehicle. TGFβ2 increased expression of SM22α and form stress fibers in the enlarged, elongated cells. ZO1 becomes redistributed to the cytoplasm.

*Tgfbr3*^*+/−*^:Immorto mice were generated as described [39] and maintained on a C57BL/6 SV129 mixed background. *Tgfbr3*^*+/+*^:Immorto and *Tgfbr3*^*−/−*^:Immorto immortalized epicardial cell lines were generated from littermates as described [39] from E11.5 embryos (Fig 1A and 1B). The original work that covered the generation of the embryonic epicardial cell lines used in these studies was carried out as approved on protocol M/13/156 (Joey Barnett, PI) by the Institutional Animal Care and Use Committee of Vanderbilt University.

### Cell culture

To maintain the immortalized state, cells were grown at 33°C in DMEM containing 10% fetal bovine serum, 100 U/ml penicillin/streptomycin, Insulin–Transferrin–Selenium (ITS: 1 μg/ml insulin, 5.5 × 10−^4^ μg/ml transferrin, 0.677 μg/ml selenium), and 10 U/ml interferon γ (INFγ). For experiments, the T-antigen was inactivated by culturing at 37°C in the absence of ITS or INFγ. Cells were seeded at 200,000 cells per well of a 6-well tissue culture plate and allowed to adhere overnight at 37°C. The following day the medium was replaced with media containing either VEH, 250 pM TGFβ1, 250 pM TGFβ2, or 5 nM BMP2. After a 72 hour incubation period at 37°C, total RNA was isolated via standard phenol-chloroform extraction (TRIzol Invitrogen). RNA was purified (Qiagen mini-prep kit) following the manufacturer’s protocol. Quantity and quality of RNA was determined by an Agilent Bioanalyzer. One well of a 6-well plate yielded 10–20 μg of RNA.

### qRT-PCR

Quantitative Real Time PCR qRT-PCR was performed as described [16]. Briefly, cDNA was generated from 1μg total RNA using oligo-dT primers and Superscript III polymerase (Invitrogen). Real-time PCR analysis was done with iQ SYBR Green Supermix (Bio-Rad) in the Bio-Rad iCycler for 40 cycles. Primer pairs forward (F) and reverse (R): *GAPDH* F-ATGACAATGAATACGGCTACAG, R-TCTCTTGCTCAGTGTCCTTG; *Mylk* F-CCAAGGACCGGATGAAGAAATA, R-CCCTGAGATCATTGCCATAGAG; *Sema3d* F-TGGGACATAGAAGCATTAG, R-AGAGGCTTGTTGGGATTTAGG; *Sxc* F-AGGGCCTATGAACAGAGAGAT, R-GTAGAGAGCCAGCATGGAAAG; *Cadm1* F-TCTGTAGGCGGCTCAGTATAG, R-CTCACATGTCGGGTCTGTTTAG; *Krt8* F-GGCCAACCTTAGGAGGAATTT, R-GAGCCAGCTGAGGCTTTATT; *Chst7* F-GTGAGACACTGGGACTGATTTG, R-GCCAAGGTGTCTGTCATTACTT; *Versican* F-CAGGCTATCACAGGCAGATTAG, R-CAGAAGCCAAGGAGTCATTCA.

### RNA-seq

The generation of RNAseq libraries without normalizations or RNA/cDNA fragmentation were performed as described [40]. Libraries were sequenced as 50bp paired end sequences on a single lane of the Illumina HiSeq2000. TOPHAT [41] (http://tophat.cbcb.umd.edu/) was used to align HiSeq 2000 reads to produce bam files. Reads were normalized to total mRNA (total aligned reads per gene-loci per million). Gene expression profiles were generated as described [42] using a Bayesian p-value (S1 and S2 Figs). Data deposited at the Cardiovascular Development Consortium (CvDC) Data Repository (https://hci-bio-app.hci.utah.edu/gnomex/), external experiment number 38R1 (https://b2b.hci.utah.edu/gnomex/gnomexGuestFlex.jsp?requestNumber=38R1).

### SEAP Reporter System

The pNF-ĸB-SEAP (Clonetech) reporter was used to determine NF-κB activity in cells as described [43, 44]. Briefly, cells were co-transfected with pNF-ĸB-SEAP and β-galactosidase expression vector (p-CMVβ) and after 24 hours incubated with ligand (250pM TGFβ1, 250 pM TGFβ2, or 5nM BMP2). 24 hours after ligand addition the supernatant was assayed for alkaline phosphatase. β-galactosidase activity was used to normalize alkaline phosphatase activity.

### Transwell Invasion Assay

Invasion assay performed using a collagen pad in a transwell as described in [16].

## Results

### Transcriptional profiles of *Tgfbr3*^*+/+*^ and *Tgfbr3*^*-/-*^ cells confirm epicardial cell identity and ligand response

**Fig 2:**
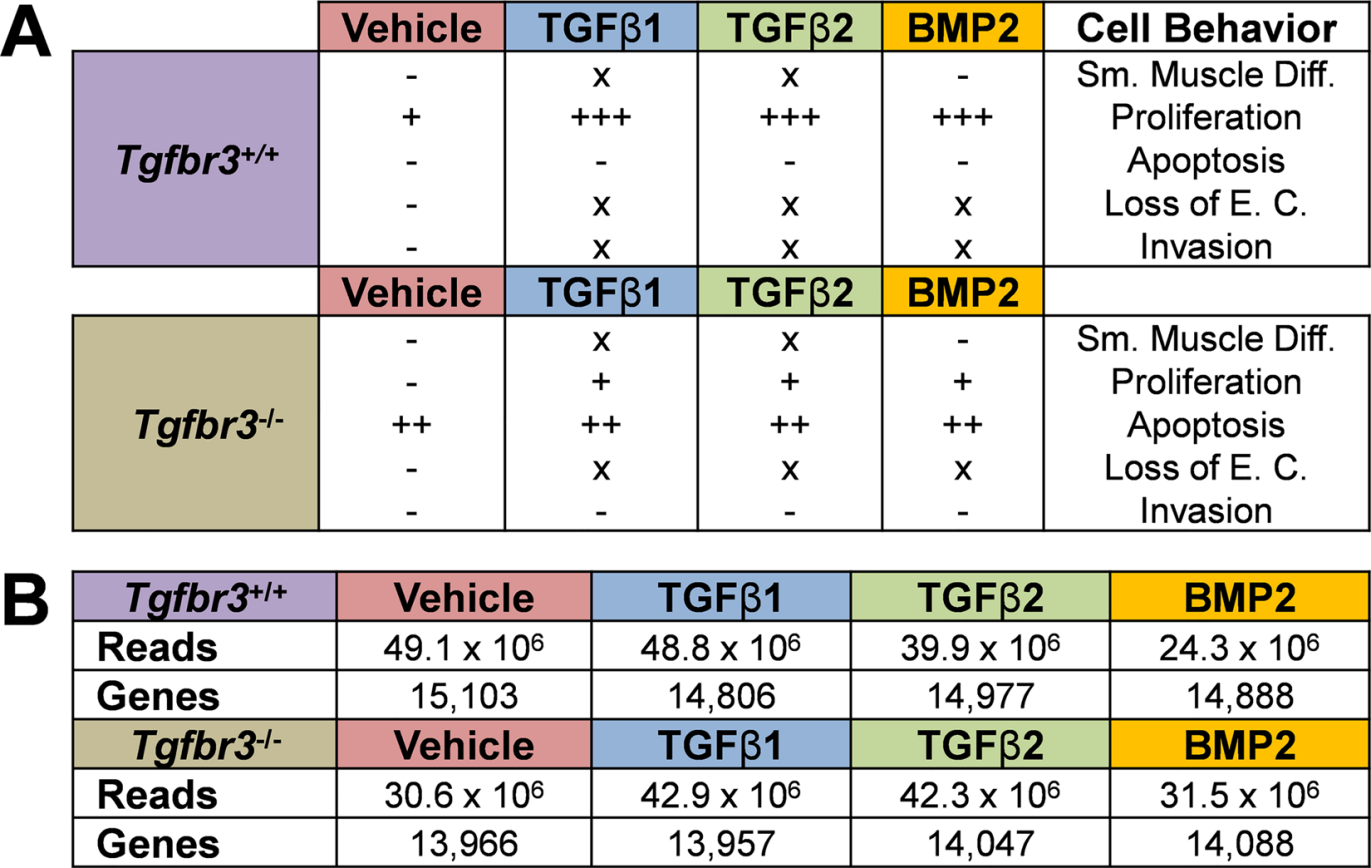
*Tgfbr3*^*-/-*^ epicardial cells have dysregulated proliferation, apoptosis, and invasion. (A) Summary of the phenotypes of *Tgfbr3*^*+/+*^ and *Tgfbr3*^*-/-*^ epicardial cells *in vitro*. EC—epithelial character, SM Diff.- smooth muscle differentiation. (B) RNA-seq analysis of *Tgfbr3*^*+/+*^ and *Tgfbr3*^*-/-*^ epicardial cells incubated with ligand for 72 hours. Reads—the total number of mapped sequences for each of the 8 groups (in duplicate). Genes—the total number of genes with a significant number of reads (>10) mapped.

**Fig 3:**
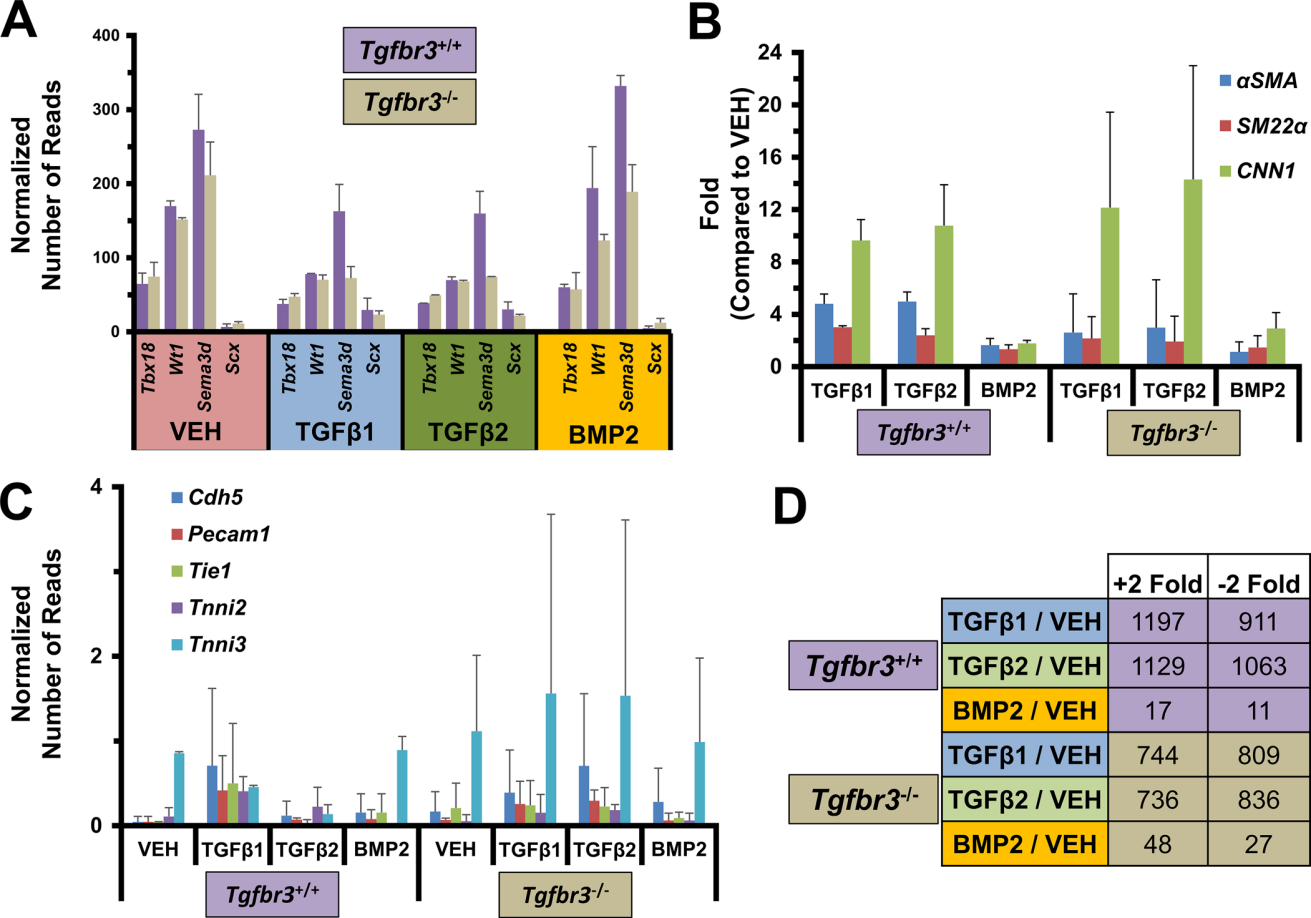
*Tgfbr3*^*+/+*^ and *Tgfbr3*^*-/-*^ epicardial RNA-seq datasets confirm cell identity and differential ligand response. (A) Cells express epicardial markers. Mean normalized reads between replicates and standard error are depicted. (B) Smooth muscle markers are markedly induced with TGFβ1 and TGFβ2 compared to BMP2 incubation. Fold is relative to VEH for each genotype. (C) Endothelial or myocardial markers are not expressed at significant levels (< 2 normalized reads). Mean normalized reads between replicates and standard error are depicted. (D) Genes >2-fold differentially expressed after ligand treatment compared to vehicle are depicted. Fewer genes are induced by incubation with TGFβ1–2 treatment in *Tgfbr3*^*-/-*^ epicardial cells compared to *Tgfbr3*^*+/+*^, while the opposite is true with BMP incubation.

We undertook a transcriptional profiling approach to examine the genes downstream of TGFβR3 in epicardial cells *in vitro*. This system was chosen since it provides defined phenotypic endpoints to contrast between genotypes and different ligand incubation groups (Fig 2A). *Tgfbr3*^*+/+*^ and *Tgfbr3*^*-/-*^ epicardial cells were incubated for 72 hours with VEH or ligands known to drive TGFβR3-dependent invasion (TGFβ1, TGFβ2, BMP2) [25]. After incubation RNA was harvested and analyzed by RNA-seq as described. More than 24 million reads were obtained for each group (VEH, TGFβ1, TGFβ2, BMP2) in each genotype (Fig 2B–Genes). Over 13,900 genes were significantly expressed (Reads >10) in each dataset (Fig 2B–Reads). Of these genes, we observed that markers of embryonic epicardial cells (*Wt1* [45], *Tbx18* [46], *Sema3d* [14], *Scx* [14]) were expressed in all data sets (Fig 3A) but markers of endothelial (*Cdh5 [47], Pecam1 [48], Tie1 [49]*) or myocardial (*Tnni2* [50, 51]*, Tnni3 [51, 52]*) lineages were not (Fig 3). *Sema3a* and *Scx* expression were confirmed with qRT-PCR (S3 Fig). The expression profile observed confirms the epicardial identity of these cells.

We have previously reported that TGFβ1 and TGFβ2 promote loss of epithelial character, invasion, and smooth muscle differentiation defined as the increased expression of the smooth muscle markers *α-Sma*, *Sm22α*, and *Cnn1* (reviewed [53]) in *Tgfbr3*^*+/+*^ and *Tgfbr3*^*-/-*^ epicardial cells (TGFβ2 depicted in Fig 1C). BMP2 promotes loss of epithelial character and invasion but not smooth muscle differentiation [26]. RNA-seq data sets demonstrated that the level of expression of *α-Sma*, *Sm22α*, and *Cnn1* were >2-fold higher in TGFβ1- and TGFβ2-incubated cells of each genotype (Fig 3B). BMP2 incubation resulted in a considerably lower induction of smooth muscle markers (Fig 3B). Hundreds of genes had >2-fold increased or decreased expression after TGFβ1 or TGFβ2 incubation (Fig 3B). Far fewer genes were >2-fold differentially expressed after BMP2 incubation (Fig 3B) which may be at least partially due to the inability of BMP2 to induce smooth muscle differentiation. Of note, fewer genes were induced with TGFβ1 or TGFβ2 incubation in *Tgfbr3*^*-/-*^ cells when compared to *Tgfbr3*^*+/+*^ cells, while the opposite was found with BMP2 incubation. This transcriptional profile of *Tgfbr3*^*+/+*^ and *Tgfbr3*^*-/-*^ epicardial cells confirms both the epicardial identity and the known response of these cells to ligand. Therefore, we used these data sets for further analysis towards delineating the downstream signaling pathways of TGFβR3 in the epicardium.

### Dysregulation of gene expression in epicardial cells lacking TGFβR3

**Fig 4:**
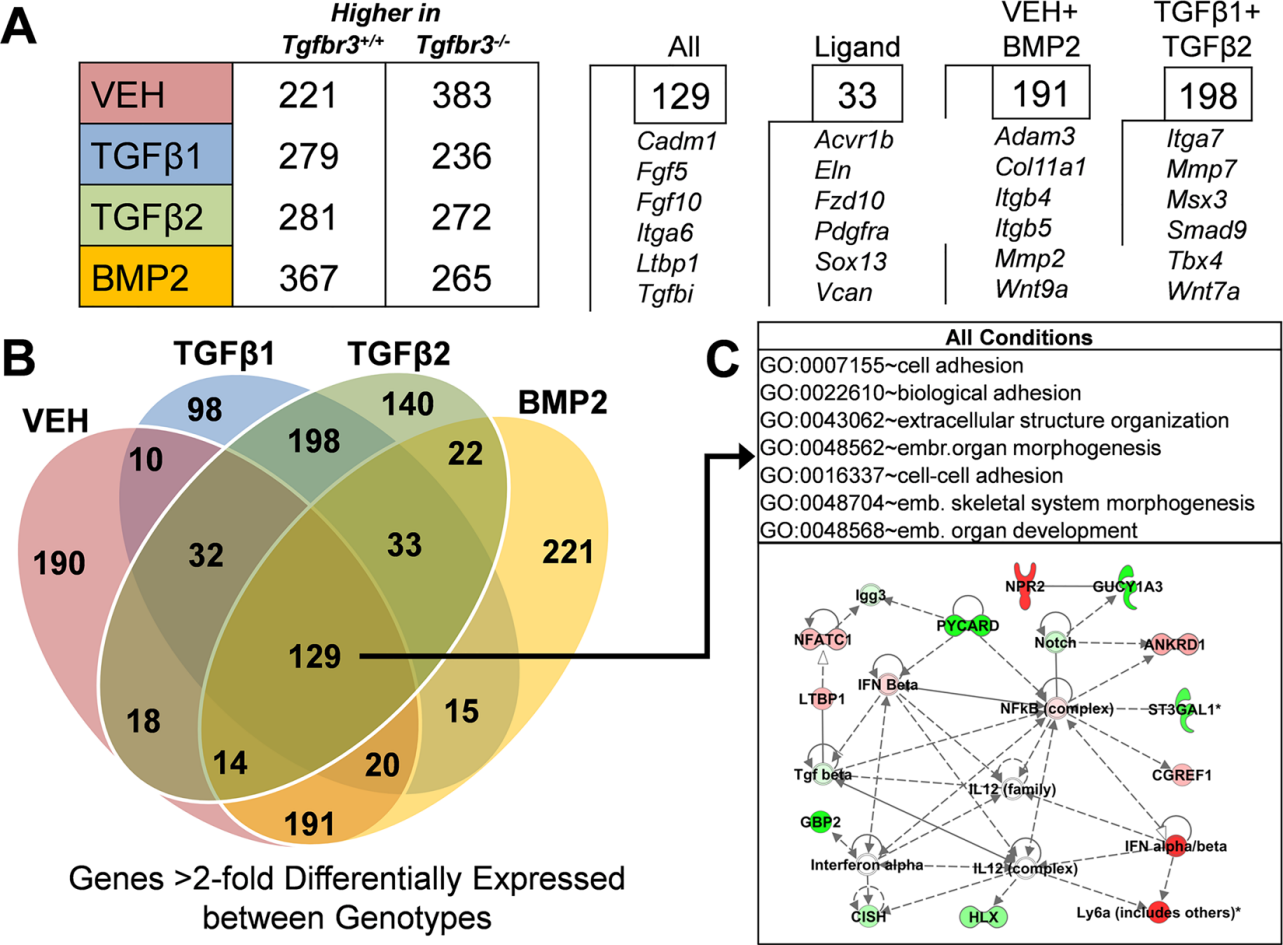
RNA-seq analysis identifies genes dysregulated in *Tgfbr3*^*-/-*^ epicardial cells. (A) (Left) The number of genes >2-fold (p<0.001) differentially expressed between *Tgfbr3*^*+/+*^ and *Tgfbr3*^*-/-*^ epicardial cells for each group. (Right) The number genes similarly dysregulated within selected groups that were also annotated in the IPA database are shown with genes found in each. (B) The number of overlapping genes >2-fold differentially regulated (p<0.001) was determined and mapped. 129 genes were similarly dysregulated across all groups. (C) (Top) Gene ontology analysis of these 129 genes by DAVID revealed a significant (p<0.0001) enrichment of genes associated with specific biological processes. emb.- embryonic. (Bottom) A representative network generated by gene regulatory network analysis of the 129 genes using Ingenuity Pathway Analysis software is depicted. Green- expressed higher in*Tgfbr3*^*+/+*^, Red- expressed higher in *Tgfbr3*^*-/-*^.

To ascertain the genes differentially regulated after the loss of *Tgfbr3*, we compared the expression profiles of *Tgfbr3*^*+/+*^ and *Tgfbr3*^*-/-*^ epicardial cells incubated with VEH, TGFβ1, TGFβ2, or BMP2. We observed hundreds of genes >2-fold (p<0.001) differentially regulated between genotypes in cells incubated with VEH (604), TGFβ1 (515), TGFβ2 (553), or BMP2 (632) (Fig 4A; S1–S4 Tables). The overlap between these >2-fold differentially expressed gene lists were plotted (Fig 4B) identifying 129 genes similarly dysregulated across all groups. This list of genes is defined as those that are differentially expressed after the loss of *Tgfbr3* regardless of ligand incubation. To gain a better understanding of the biological processes these genes may be associated with, Gene Ontology (GO) analysis was undertaken using Database for Annotation, Visualization, & Integrated Discovery (DAVID) software [54]. GO analysis identified enriched biological processes (p<0.0001) associated with cell adhesion and extracellular structure organization indicating a potential defect in cell interaction with the ECM, a vital component of cell invasion (Fig 4C–Top). In order to understand how these genes may interact, Ingenuity Pathway Analysis (IPA) software (www.ingenuity.com) was used to perform Gene Regulatory Network (GRN) analysis. An example network is depicted (Fig 4C–Bottom) which revealed TGFβ and Notch signaling pathways [55], both known important regulators of epicardial cell behavior and subsequent coronary vessel development. We also identified signaling pathways previously unexamined in epicardial development. For example, NF-ĸB signaling emerged as a central node in this analysis providing a candidate for further evaluation.

To gain a more detailed understanding of the genes dysregulated after loss of TGFβR3, we examined genes with dysregulated expression in specific ligand incubation groups. When considering the overlap between genes in at least any two groups (VEH, TGFβ1, TGFβ2, or BMP2) that are similarly >2-fold differentially expressed between genotypes (Fig 4B), we observed that there are many more genes shared between TGFβ1-TGFβ2 (198) and VEH-BMP2 (191) than any other comparison (for example; VEH-TGFβ1 (10), BMP2-TGFβ2 (22)). This may reflect the fact that both TGFβ1 and TGFβ2 induce smooth muscle differentiation. GO analysis of the 198 genes uniquely dysregulated in *Tgfbr3*^*-/-*^ cells after TGFβ1 and TGFβ2 incubation identified vasculature development as the most enriched biological process (p<0.001) (Table 1). This analysis is consistent with altered vascular development in the epicardium after loss of *Tgfbr3*. However, processes associated with vascular development were not found to be significantly enriched by GO analysis in the 191 genes uniquely >2-fold dysregulated between genotypes with VEH and BMP2 incubation or in the 221 genes uniquely dysregulated with BMP2 incubation (Table 1).

**Table 1:** GO Analysis of Genes >2-fold Differentially Expressed Between Genotypes Unique to Specific Ligand Incubation Groups.

GO Term	p-value
**TGFβ1 + TGFβ2**	
GO:0001944~vasculature development	3.71E-04
GO:0000122~negative regulation of transcription from RNA polymerase II promoter	0.001014
GO:0032963~collagen metabolic process	0.001046
GO:0006357~regulation of transcription from RNA polymerase II promoter	0.001059
GO:0001525~angiogenesis	0.001153
**VEH + BMP2**	
GO:0022037~metencephalon development	0.017354
GO:0050900~leukocyte migration	0.018991
GO:0030902~hindbrain development	0.043128
GO:0042127~regulation of cell proliferation	0.048931
GO:0008284~positive regulation of cell proliferation	0.051412
**BMP2**	
GO:0007242~intracellular signaling cascade	1.63E-04
GO:0009069~serine family amino acid metabolic process	0.001428
GO:0006534~cysteine metabolic process	0.002140
GO:0007188~G-protein signaling, coupled to cAMP nucleotide second messenger	0.002451
GO:0030534~adult behavior	0.002719

**Fig 5:**
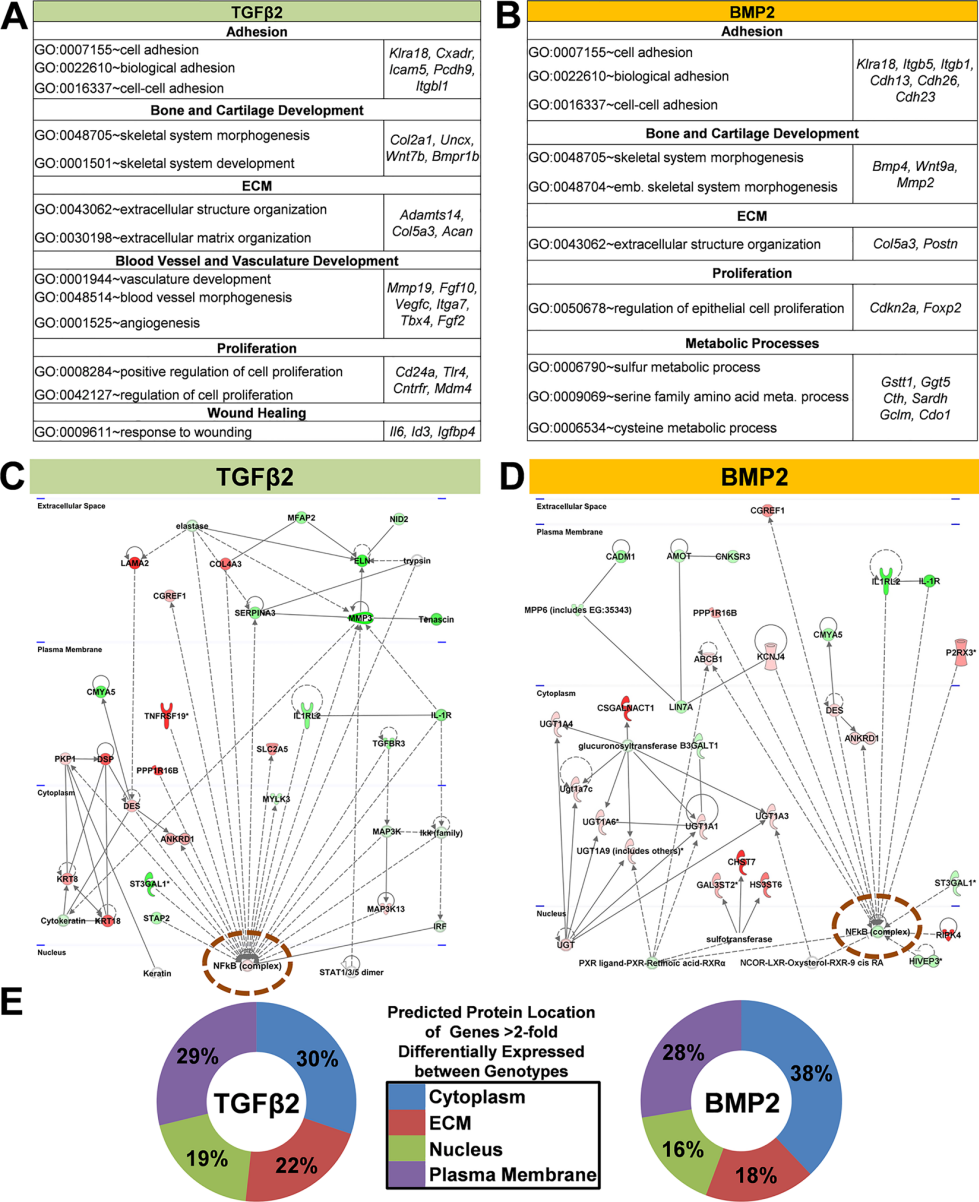
Gene regulatory network analysis identifies NF-kB signaling as a central node. Genes >2-fold (p<0.001) differentially expressed between *Tgfbr3*^*+/+*^ and *Tgfbr3*^*-/-*^ epicardial cells incubated with either TGFβ2 (A) or BMP2 (B) were subjected to gene ontology analysis (using DAVID software, p<0.0001). (C-D) NF-ĸB signaling (orange circle) is a central node in representative networks generated by gene regulatory network analysis (using Ingenuity Pathway Analysis software). Green- expressed higher in*Tgfbr3*^*+/+*^, Red- expressed higher in *Tgfbr3*^*-/-*^. (E) The distribution of the predicted protein location in the cell is depicted (proteins with unknown location are not shown).

Although we have reported that TGFβ2 promotes loss of epithelial character and smooth muscle differentiation via ALK5 signaling and BMP2 promotes only the loss of epithelial character via ALK3 signaling, both ligands require TGFβR3 to mediate invasion [26]. To gain a better understanding of how TGFβ and BMP signaling are impacted by the loss of *Tgfbr3*, we examined genes >2-fold differentially expressed between *Tgfbr3*^*+/+*^ and *Tgfbr3*^*-/-*^ epicardial cells incubated with TGFβ2 or BMP2. GO analysis identified that biological processes associated with blood vessel development and angiogenesis were enriched (p<0.0001) in TGFβ2 but not BMP2 gene lists (genes including *Fgf2* and *Vegfc*) (Fig 5A). Thus, while TGFβ induces smooth muscle differentiation in *Tgfbr3*^*-/-*^ cells, there remain defects in the signaling networks associated with formation of the vasculature. Biological processes enriched in both of these TGFβ2 and BMP2 gene lists include processes associated with cell adhesion, extracellular matrix (ECM) organization, and proliferation (Fig 5A and 5B). These results are consistent with the known epicardial phenotype of *Tgfbr3*^*-/-*^ embryos [15, 16].

**Fig 6:**
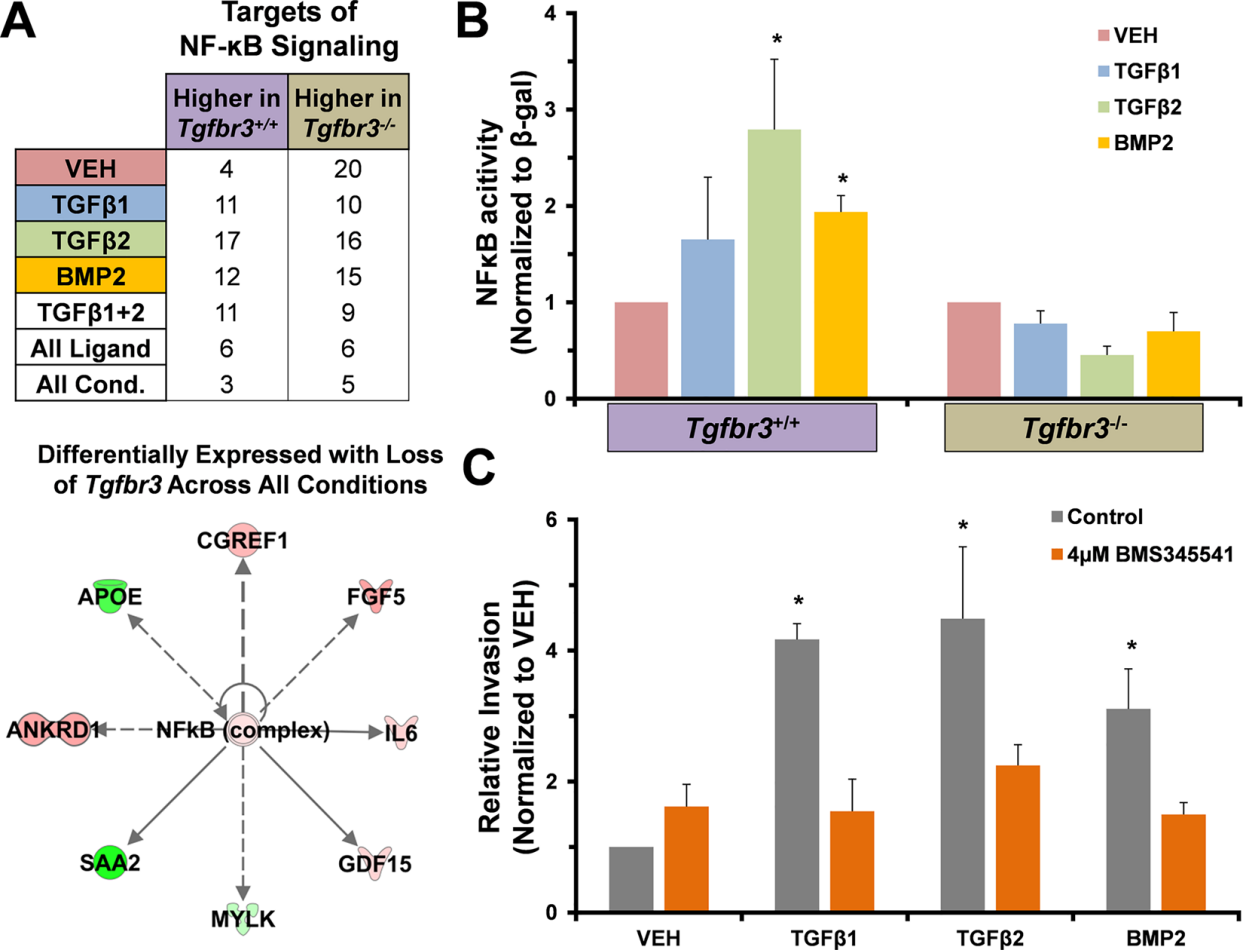
*Tgfbr3*^*-/-*^ epicardial cells fail to activate the NF-ĸB signaling pathway. (A) (TOP) Genes dysregulated in each group (>2-fold, p<0.001) were counted. (BOTTOM) Shared targets of NF-ĸB signaling dyregulated in all groups are shown. Red**—**expressed higher in*Tgfbr3*^*+/+*^, Green**—**expressed higher in *Tgfbr3*^*-/-*^. (B) Cells transfected with an NF-ĸB responsive SEAP reporter construct and incubated with VEH, TGFβ1, TGFβ2, or BMP2 revealed the inability of *Tgfbr3*^*-/-*^ cells to induce NF-ĸB signaling. (C) Incubation of *Tgfbr3*^*+/+*^ epicardial cells in a transwell invasion assay with an NF-ĸB inhibitor (BMS345541) significantly reduced invasion (* = p < .01) in response to ligands known to promote *Tgfbr3*-dependent invasion.

To reveal interactions, genes >2-fold differentially expressed between genotypes after TGFβ2 or BMP2 incubation were used to generated GRNs using IPA software. Example networks are depicted in Fig 5C and 5D. The TGFβ2 network features predicted proteins known to be located in the ECM that regulate cell-ECM interactions. Several of these genes have lower levels of expression in *Tgfbr3*^*-/-*^ cells compared to *Tgfbr3*^*+/+*^ cells (green nodes). Also present are several genes encoding cytoplasm and plasma membrane proteins that are expressed at higher levels in *Tgfbr3*^*-/-*^ epicardial cells compared to *Tgfbr3*^*+/+*^ cells (red nodes). These genes are associated with epithelial sheet stability and adhesion, for example *Krt18* and *Krt8*, whose expression pattern was confirmed using qRT-PCR (S3 Fig). The BMP2 network also features genes that are expressed at higher levels in *Tgfbr3*^*+/+*^ cells whose proteins are known to associate with the plasma membrane to regulate cell adhesion and cell migration (*Lin7a* [56], *Amot* [57]). Nodes associated with ECM protein synthesis (*Csgalnact1* [58]) or post translation modification of receptors that interact with ECM (*Chst7* [59]) were also observed in the BMP2 network and the induction of Chst7 in immortalized epicardial cell was confirmed using qRT-PCR (S3 Fig). These networks indicate a deficit in the ability of cells to interact with the ECM and a potential defect in cell motility. NF-ĸB was a central node in not only the TGFβ2 and BMP2 networks (Fig 5C and 5D–Orange circle), but also in GRNs derived from genes differentially expressed between genotypes with VEH or TGFβ1 incubation (S4 Fig). We identified several genes known to be downstream of NF-κB signaling that were differentially regulated in each ligand incubation group (S5A–S5D Fig) when compared between genotypes. A table depicting the overlap between these genes is shown (Fig 6A–Top). GRN analysis indicates that NF-ĸB signaling may be dysregulated with loss of TGFβR3 in epicardial cells. The dysregulated NF-ĸB signaling in both TGFβ2 and BMP2 gene lists, where a common phenotype is loss of invasion, suggests that NF-ĸB signaling may regulate cell invasion in response to these ligands.

### NF-ĸB signaling is dysregulated in *Tgfbr3*^*-/-*^ epicardial cells *in vitro*

To test the hypothesis that TGFβR3 promotes NF-ĸB signaling to regulate epicardial cell invasion we examined if NF-ĸB activity was induced by TGFβ2 or BMP2 ligand incubation in epicardial cells *in vitro* (Fig 6B). Immortalized epicardial cells incubated with TGFβ2 or BMP2 increased NF-ĸB activity compared to VEH in *Tgfbr3*^*+/+*^ epicardial cells as described [44]. TGFβ2 or BMP2 ligand incubation failed to induce NF-ĸB activity in *Tgfbr3*^*-/-*^ cells (Fig 6B). To determine if NF-ĸB signaling was required for epicardial cell invasion *in vitro*, we performed a collagen pad, transwell invasion assay with TGFβ1, TGFβ2, and BMP2 in the presence or absence of the NF-κB inhibitor, BMS345541. BMS345541 (10 μm) significantly decreased TGFβ1-, TGFβ2-, or BMP2-induced invasion in *Tgfbr3*^*+/+*^ cells when compared to VEH (Fig 6C). Together these data demonstrate that NF-ĸB signaling is dysregulated in *Tgfbr3*^*-/-*^ epicardial cells and that NF-ĸB is required for epicardial cell invasion *in vitro*. These data support the hypothesis that TGFβR3 promotes NF-ĸB activity to regulate epicardial cell invasion.

## Discussion

### Transcriptional profiling of epicardial cells

We developed a transcriptional profiling strategy using immortalized, embryonic epicardial cells *in vitro* to identify genes and signaling pathways downstream of TGFβR3 that regulate cell invasion. Previous studies have profiled gene expression in adult epicardial cells [60, 61], the proepicardium [62], and primary epicardial cells (E12.5) [63] using microarrays, but a comprehensive transcriptional profiling of embryonic epicardial cells has been lacking. Additionally, our choice of this system allows for a first systematic examination of the genes and signaling pathways regulated by TGFβR3.

### *Tgfbr3*^*-/-*^ epicardial cells have altered expression of ECM associated genes

GO and GRN analysis of genes whose expression was >2-fold dyregulated between *Tgfbr3*^*+/+*^ and *Tgfbr3*^*-/-*^ cells for each ligand incubation group revealed biological processes associated with ECM production, ECM binding, cell adhesion, and invasion. The dysregulation of gene expression associated with these processes is consistent with the known defects identified after loss of *Tgfbr3 in vivo* and *in vitro*. Epicardial cell abnormalities in *Tgfbr3*^*-/-*^ embryos include expansion of the subepicardial space and a hyperplastic, irregular epicardium, both of which suggest defects in epicardial cell interactions with the ECM [15]. Invasion of epicardial cells is also defective *in vivo* and *in vitro* in cells lacking TGFβR3 [16]. Consistent with a defect in cell interaction with the ECM, we observe that epicardial cells *in vitro* fail to invade in response to high molecular weight HA [16, 64], a major ECM component of the subepicardial space [65]. CD44 is the cell surface receptor which binds HA and this interaction is important for epicardial invasion [44]. Upregulated expression of the chondroitin sulfotransferase, *Chst7*, is associated with increased chondroitin sulfation of CD44 and decreased CD44-HA binding in multiple cell types [59, 66, 67]. *Chst7* had markedly increased expression (>4-fold) in *Tgfbr3*^*-/-*^ cells when compared to *Tgfbr3*^*+/+*^ cells across all ligand incubation groups. These data suggest that the inability of *Tgfbr3*^*-/-*^ cells to undergo invasion in respond to HA may result from increased chondroitin sulfation of CD44.

The myocardium and proepicardium both contribute to the ECM contained in the subepicardial space [65, 68], yet the exact makeup and source is unknown. *Tgfbr3*^*-/-*^ epicardial cells show dysregulated expression of genes encoding proteins found in the ECM, suggesting that epicardial contributions to the ECM are altered after loss of TGFβR3. *Mgp*, *Eln*, and *Tnc* have decreased expression in *Tgfbr3*^*-/-*^ cells when compared to *Tgfbr3*^*+/+*^ cells, while *Matn4* and *Emilin1* have increased expression irrespective of ligand. Alterations in the expression of specific genes were also found to be ligand-specific. *Versican* is an ECM component contained in the supepicardial space [69] that promotes cell invasion in some cancer cells (reviewed [70]) and is required for endocardial cushion formation and subsequent EMT [71, 72]. *Versican* has >2-fold higher expression after ligand (TGFβ1, TGFβ2, BMP2) incubation when compared to VEH in *Tgfbr3*^*+/+*^ cells. Ligand induction of *Versican* expression is decreased in *Tgfbr3*^*-/-*^ cells (S3 Fig), demonstrating that *Versican* expression is dependent on *Tgfbr3*-ligand interaction. Together, these data suggest the defects in coronary vessel development are due to both the altered response to, and expression of, ECM components by epicardial cells following the loss of *Tgfbr3*.

### TGFβ- and BMP-mediated gene expression programs are dysregulated in *Tgfbr3*^*-/-*^ epicardial cells

Distinct differences were observed in dysregulated gene expression between epicardial cells incubated with BMP and TGFβ ligands after loss of TGFβR3. Analysis of the genes dysregulated between *Tgfbr3*^*+/+*^ and *Tgfbr3*^*-/-*^ epicardial cells revealed potentially different mechanisms between BMP2 and TGFβ1 or TGFβ2 mediated-GRNs that may underlay a defect in cell invasion. BMP2 is important in the specification and maintenance of proepicardial cell identity [73], directed proepicardial cell migration [74], and epicardial cell loss of epithelial character and invasion [25]. GRNs generated from the genes dysregulated between genotypes after BMP2 incubation revealed a grouping of genes encoding PDZ domain-containing proteins that had decreased expression in *Tgfbr3*^*-/-*^ epicardial cells when compared to *Tgfbr3*^*+/+*^ cells (*Amot*, *Cadm1*, *Cnksr3*, *Lin7a*, *Mpp6*). Several of these genes (*Amot* [57, 75, 76], *Cadm1* [77], *Cnksr3* [78], *Lin7a* [56, 79]) have been previously reported to promote cell migration but a role in the epicardium has not been described. These observations are consistent with the known role in BMP2 in directing epicardial migration and the decrease of invasion observed in *Tgfbr3*^*-/-*^ epicardial cells. These data also provide an intriguing set of candidate genes as the PDZ domain of TGFβR3 and a protein that interacts with this domain, GIPC, are required for TGFβR3-mediated invasion *in vitro* [16]. GRNs generated from the genes dysregulated between genotypes after TGFβ1 or TGFβ2 incubation had different features from the BMP2 network. A large grouping of genes whose expression was reduced after loss of TGFβR3 was localized to the extracellular space in the TGFβ1 and TGFβ2 networks. These genes were involved in the production of ECM components (*Eln*), matrix degradation (*Mmp3*, *Elastase*), and ECM organization (*Mfap2*). A different grouping of genes expressed at higher levels after loss of TGFβR3 localized to the cytoplasm were associated with epithelial sheet stability and non-motile cells (*Krt8*, *Krt18*). The GRNs are consistent with a population of cells with dysregulated ECM interaction and reduced motility. In addition, genes in signaling pathways associated with vascular development and angiogenesis were dysregulated between genotypes with TGFβ1 and TGFβ2 but not BMP2 incubation. This finding is particularly interesting as factors secreted by the epicardium after injury to the heart are hypothesized to promote the formation of new vessels in the impacted area [80]. To support proper coronary vessel development signaling events must be tightly regulated in the epicardium *in vivo*. Our data demonstrates TGFβR3 is an important component of the regulatory machinery that integrates TGFβ and BMP signaling in epicardial cells.

### Loss of TGFβR3 disrupts NF-κB signaling in embryonic epicardium

TGFβR3 is required for invasion promoted by TGFβ1, TGFβ2 and BMP2, suggesting that a TGFβR3-dependent signaling mechanism that regulates invasion is shared between these ligands. Our data predicts NF-ĸB signaling is dysregulated in *Tgfbr3*^*-/-*^ epicardial cells. GRN’s generated from genes >2-fold differentially expressed between *Tgfbr3*^*+/+*^ and *Tgfbr3*^*-/-*^ epicardial cells across each ligand incubation group (VEH, TGFβ1, TGFβ2, BMP2) identified NF-ĸB signaling as a central node. In support of a role for NF-ĸB signaling, genes known to be regulated directly or indirectly downstream of NF-ĸB were also dysregulated. Incubation of epicardial cells with TGFβ2 or BMP2 increased NF-ĸB activity in *Tgfbr3*^*+/+*^ but not in *Tgfbr3*^*-/-*^ cells, demonstrating that TGFβR3 is required for NF-ĸB activity in epicardial cells. Several mechanisms may account for the ability of TGFβR3 to regulate NF-ĸB signaling. Previous studies have found that TGFβR3 can suppress NF-ĸB signaling via interaction with β-arrestin2 [81]. IL-1β, an upstream regulator of NF-ĸB signaling, can suppress TGFβR3 signaling by binding to TRAF6 which subsequently sequesters TGFβR3 from TGFβR2 [82]. Here, reduced NF-ĸB activity may result from a >2-fold reduction in the expression of an important upstream regulator of NF-ĸB signaling, *Il-1r* (reviewed [83]), in *Tgfbr3*^*-/-*^ cells when compared to *Tgfbr3*^*+/+*^ cells. Reduced NF-ĸB activity may also result from decreased expression in *Tgfbr3*^*-/-*^ cells of Myosin Light Chain Kinase (*Mylk*) as seen in RNA-seq (S1–S4 Tables) and by qRT-PCR (S3 Fig). MYLK has recently been shown to promote activation of NF-ĸB signaling [84]. MYLK kinase activity is required for MyD88 and IRAK4 complex formation, which in turn is required to activate NF-ĸB downstream of lipopolysaccharide [85, 86], in lung endothelial cells [84]. Given the known roles of *Mylk* in regulating smooth muscle behavior [87], cell migration [88], and a link to coronary artery disease [89], the elucidation of the regulatory interactions between MYLK, TGFβR3, and NF-ĸB in epicardial cells may provide key insights into coronary vessel development.

While TGFβR3 signaling has been previously reported to both inhibit [81, 90] and promote [91] NF-ĸB signaling, a consistent fact in all of these studies is that a decrease in NF-ĸB activity was coincident with decreased invasion. Here we used a small molecule inhibitor and showed that NF-ĸB activity was required for epicardial cell invasion. In a recent, separate study [92], we confirmed that invasion is dependent upon NFκB signaling and that *Tgfbr3*^*-/-*^ cells lack both invasion and NFκB activation. Overexpression of TGFβR3 in *Tgfbr3*^*-/-*^ cells rescues ligand-dependent invasion that is sensitive to NFκB inhibitors. Further, endocardial cell invasion, a TGFβR3-dependent process [93], is decreased by the inhibition of NFκB activity. These data suggest that NF-ĸB is a shared signaling pathway downstream of ligand and that TGFβR3 interaction is required for cell invasion. Therefore, we propose that the disruption of TGFβR3 regulated NF-ĸB signaling is a mechanism responsible for the loss of invasion in epicardial cells and ultimately failed coronary vessel development in *Tgfbr3*^*-/-*^ embryos.

## Supporting Information

S1 Fig Variability of RNA-seq data sets. The reads for the two biological replicates (n = 1, n = 2) for each group (VEH, TGFβ1, TGFβ2, BMP2) in *Tgfbr3*^*+/+*^ (A-D) or *Tgfbr3*^*-/-*^ (E-H) were plotted against each other. There was a high degree of agreement in *Tgfbr3*^*+/+*^ (A-D) (R>0.87) or *Tgfbr3*^*-/-*^ (E-H) (R>0.89) datasets. These comparisons support a high degree of agreement between biological replicates.
(TIF)

S2 Fig Comparison of differential gene expression between biological replicates. Plots mapping the fold (log base 2) difference >2-fold in expression between VEH and ligand incubated groups in *Tgfbr3*^*+/+*^ (A) or *Tgfbr3*^*-/-*^ (B) in biological replicates (X-axis: n = 1, Y-axis: n = 2) shown. Genes that have agreement, defined as having >2-fold (p<0.001) increased or decreased expression in a specific comparison in both replicates, are mapped to quadrants I (upper right) or III (lower left) of a plot. Genes that show disagreement, defined as having >2-fold (p<0.001) increased expression in a tissue in one replicate and decreased in another (or vis versa), are mapped to quadrants II (upper left) or IV (lower right). There was a high degree of agreement in *Tgfbr3*^*+/+*^ (A) (R>0.85) or *Tgfbr3*^*-/-*^ (B) (R>0.89) datasets across all comparisons [94]. Variability between biological replicates was determined.
(PDF)

S3 Fig Genes with dysregulated expression in *Tgfbr3*^*-/-*^ epicardial cells. Differential gene expression between *Tgfbr3*^*+/+*^ and *Tgfbr3*^*-/-*^ epicardial cells observed in RNA-seq data was evaluated using qRT-PCR analysis (n = 3). Expression was normalized to the constitutive expression level of GAPDH RNA and the ratio of transcriptional abundance found in *Tgfbr*^*+/+*^ to *Tgfbr*^*-/-*^ is depicted.
(TIF)

S4 Fig Gene regulatory network analysis identifies NF-ĸB signaling as a central node. Genes >2-fold (p<0.001) differentially expressed between *Tgfbr3*^*+/+*^ and *Tgfbr3*^*-/-*^ epicardial cells incubated with either TGFβ1 (A) or VEH (B) were subjected (A) to gene ontology analysis (using DAVID software, p<0.0001). (C-D) NF-ĸB signaling (orange circle) is a central node in representative networks generated by gene regulatory network analysis (using Ingenuity Pathway Analysis software). Green- expressed higher in*Tgfbr3*^*+/+*^, Red- expressed higher in *Tgfbr3*^*-/-*^.
(PDF)

S5 Fig Genes downstream of NF-ĸB signaling dysregulated with loss of *Tgfbr3* in epicardial cells *in vitro*. Genes identified as being >2-fold differentially regulated between *Tgfbr3*^*+/+*^ or *Tgfbr3*^-/-^ epicardial cells incubated with (A) VEH, (B) BMP2, (C) TGFβ1, or (D) TGFβ2. Solid lines denote a direct interaction while dotted lines denote indirect interaction between proteins. Green- higher expression in *Tgfbr3*^*+/+*^. Red- higher expression in *Tgfbr3*^-/-^.
(PDF)

S1 Table Genes >2-fold dysregulated between *Tgfbr3*^*+/+*^ and *Tgfbr3*^*-/-*^ epicardial cells after VEH incubation. Genes identified as being >2-fold differentially regulated between *Tgfbr3*^*+/+*^ or *Tgfbr3*^-/-^ epicardial cells incubated with VEH. Genes listed in descending order of significance for each of 2 biological replicates. For each gene, p-value, location in cell, and function are listed.
(XLSX)

S2 Table Genes >2-fold dysregulated between *Tgfbr3*^*+/+*^ and *Tgfbr3*^*-/-*^ epicardial cells after TGFβ1 incubation. Genes identified as being >2-fold differentially regulated between *Tgfbr3*^*+/+*^ or *Tgfbr3*^-/-^ epicardial cells incubated with TGFβ1. Genes listed in descending order of significance for each of 2 biological replicates. For each gene, p-value, location in cell, and function are listed.
(XLSX)

S3 Table Genes >2-fold dysregulated between *Tgfbr3*^*+/+*^ and *Tgfbr3*^*-/-*^ epicardial cells after TGFβ2 incubation. Genes identified as being >2-fold differentially regulated between *Tgfbr3*^*+/+*^ or *Tgfbr3*^-/-^ epicardial cells incubated with TGFβ2. Genes listed in descending order of significance for each of 2 biological replicates. For each gene, p-value, location in cell, and function are listed.
(XLSX)

S4 Table Genes >2-fold dysregulated between *Tgfbr3*^*+/+*^ and *Tgfbr3*^*-/-*^ epicardial cells after BMP2 incubation. Genes identified as being >2-fold differentially regulated between *Tgfbr3*^*+/+*^ or *Tgfbr3*^-/-^ epicardial cells incubated with BMP2. Genes listed in descending order of significance for each of 2 biological replicates. For each gene, p-value, location in cell, and function are listed.
(XLSX)

## References

[1] Olivey HE, Compton LA, Barnett JV. Coronary vessel development: the epicardium delivers. Trends in cardiovascular medicine. 2004;14(6):247–51. Epub 2004/09/29. 10.1016/j.tcm.2004.07.001 . 15451517

[2] Olivey HE, Svensson EC. Epicardial-myocardial signaling directing coronary vasculogenesis. Circulation research. 2010;106(5):818–32. Epub 2010/03/20. 10.1161/CIRCRESAHA.109.209197 20299672 PMC2843003

[3] Tian X, Pu WT, Zhou B. Cellular origin and developmental program of coronary angiogenesis. Circulation research. 2015;116(3):515–30. 10.1161/CIRCRESAHA.116.305097 . 25634974 PMC6914229

[4] Manner J. Experimental study on the formation of the epicardium in chick embryos. Anat Embryol (Berl). 1993;187(3):281–9. Epub 1993/03/01. . 8470828 10.1007/BF00195766

[5] Viragh S, Challice CE. The origin of the epicardium and the embryonic myocardial circulation in the mouse. Anat Rec. 1981;201(1):157–68. Epub 1981/09/01. 10.1002/ar.1092010117 . 7305017

[6] von Gise A, Pu WT. Endocardial and epicardial epithelial to mesenchymal transitions in heart development and disease. Circulation research. 2012;110(12):1628–45. Epub 2012/06/09. 10.1161/CIRCRESAHA.111.259960 22679138 PMC3427736

[7] Christoffels VM, Grieskamp T, Norden J, Mommersteeg MT, Rudat C, Kispert A. Tbx18 and the fate of epicardial progenitors. Nature. 2009;458(7240):E8–9; discussion E-10. Epub 2009/04/17. 10.1038/nature07916 . 19369973

[8] Gittenberger-de Groot AC, Vrancken Peeters MP, Mentink MM, Gourdie RG, Poelmann RE. Epicardium-derived cells contribute a novel population to the myocardial wall and the atrioventricular cushions. Circulation research. 1998;82(10):1043–52. Epub 1998/06/11. . 9622157 10.1161/01.res.82.10.1043

[9] Grieskamp T, Rudat C, Ludtke TH, Norden J, Kispert A. Notch signaling regulates smooth muscle differentiation of epicardium-derived cells. Circulation research. 2011;108(7):813–23. Epub 2011/01/22. 10.1161/CIRCRESAHA.110.228809 . 21252157

[10] Lie-Venema H, Eralp I, Markwald RR, van den Akker NM, Wijffels MC, Kolditz DP, et al Periostin expression by epicardium-derived cells is involved in the development of the atrioventricular valves and fibrous heart skeleton. Differentiation. 2008;76(7):809–19. Epub 2008/02/26. 10.1111/j.1432-0436.2007.00262.x . 18294225

[11] Poelmann RE, Gittenberger-de Groot AC, Mentink MM, Bokenkamp R, Hogers B. Development of the cardiac coronary vascular endothelium, studied with antiendothelial antibodies, in chicken-quail chimeras. Circulation research. 1993;73(3):559–68. Epub 1993/09/01. . 8348697 10.1161/01.res.73.3.559

[12] Guadix JA, Carmona R, Munoz-Chapuli R, Perez-Pomares JM. In vivo and in vitro analysis of the vasculogenic potential of avian proepicardial and epicardial cells. Dev Dyn. 2006;235(4):1014–26. 10.1002/dvdy.20685 . 16456846

[13] Nesbitt TL, Patel PA, Yost MJ, Goodwin RL, Potts JD. A 3-D model of coronary vessel development. In Vitro Cell Dev Biol Anim. 2007;43(1):10–6. 10.1007/s11626-006-9007-z . 17570028

[14] Katz TC, Singh MK, Degenhardt K, Rivera-Feliciano J, Johnson RL, Epstein JA, et al Distinct compartments of the proepicardial organ give rise to coronary vascular endothelial cells. Dev Cell. 2012;22(3):639–50. 10.1016/j.devcel.2012.01.012 22421048 PMC3306604

[15] Compton LA, Potash DA, Brown CB, Barnett JV. Coronary vessel development is dependent on the type III transforming growth factor beta receptor. Circulation research. 2007;101(8):784–91. Epub 2007/08/21. 10.1161/CIRCRESAHA.107.152082 . 17704211

[16] Sanchez NS, Hill CR, Love JD, Soslow JH, Craig E, Austin AF, et al The cytoplasmic domain of TGFbetaR3 through its interaction with the scaffolding protein, GIPC, directs epicardial cell behavior. Developmental biology. 2011;358(2):331–43. Epub 2011/08/30. 10.1016/j.ydbio.2011.08.008 21871877 PMC3183347

[17] Lopez-Casillas F, Wrana JL, Massague J. Betaglycan presents ligand to the TGF beta signaling receptor. Cell. 1993;73(7):1435–44. Epub 1993/07/02. 10.1016/0092-8674(93)(0368-Z . 8391934

[18] Lopez-Casillas F, Cheifetz S, Doody J, Andres JL, Lane WS, Massague J. Structure and expression of the membrane proteoglycan betaglycan, a component of the TGF-beta receptor system. Cell. 1991;67(4):785–95. Epub 1991/11/15. 10.1016/0092-8674(91)90073-8 . 1657406

[19] Kirkbride KC, Townsend TA, Bruinsma MW, Barnett JV, Blobe GC. Bone morphogenetic proteins signal through the transforming growth factor-beta type III receptor. The Journal of biological chemistry. 2008;283(12):7628–37. Epub 2008/01/11. 10.1074/jbc.M704883200 . 18184661

[20] Wiater E, Harrison CA, Lewis KA, Gray PC, Vale WW. Identification of distinct inhibin and transforming growth factor beta-binding sites on betaglycan: functional separation of betaglycan co-receptor actions. The Journal of biological chemistry. 2006;281(25):17011–22. Epub 2006/04/20. 10.1074/jbc.M601459200 . 16621788

[21] Derynck R, Zhang YE. Smad-dependent and Smad-independent pathways in TGF-beta family signalling. Nature. 2003;425(6958):577–84. Epub 2003/10/10. 10.1038/nature02006 . 14534577

[22] Blobe GC, Schiemann WP, Pepin MC, Beauchemin M, Moustakas A, Lodish HF, et al Functional roles for the cytoplasmic domain of the type III transforming growth factor beta receptor in regulating transforming growth factor beta signaling. The Journal of biological chemistry. 2001;276(27):24627–37. Epub 2001/04/27. 10.1074/jbc.M100188200 . 11323414

[23] Blobe GC, Liu X, Fang SJ, How T, Lodish HF. A novel mechanism for regulating transforming growth factor beta (TGF-beta) signaling. Functional modulation of type III TGF-beta receptor expression through interaction with the PDZ domain protein, GIPC. The Journal of biological chemistry. 2001;276(43):39608–17. Epub 2001/09/08. 10.1074/jbc.M106831200 . 11546783

[24] Kirkbride KC, Townsend TA, Bruinsma MW, Barnett JV, Blobe GC. Bone morphogenetic proteins signal through the transforming growth factor-beta type III receptor. J Biol Chem. 2008;283(12):7628–37. Epub 2008/01/11. 10.1074/jbc.M704883200 . 18184661

[25] Sanchez NS, Barnett JV. TGFbeta and BMP-2 regulate epicardial cell invasion via TGFbetaR3 activation of the Par6/Smurf1/RhoA pathway. Cellular signalling. 2012;24(2):539–48. Epub 2011/10/29. 10.1016/j.cellsig.2011.10.006 22033038 PMC3237859

[26] Hill CR, Sanchez NS, Love JD, Arrieta JA, Hong CC, Brown CB, et al BMP2 signals loss of epithelial character in epicardial cells but requires the Type III TGFbeta receptor to promote invasion. Cellular signalling. 2012;24(5):1012–22. Epub 2012/01/13. 10.1016/j.cellsig.2011.12.022 22237159 PMC3288519

[27] Tomanek RJ, Sandra A, Zheng W, Brock T, Bjercke RJ, Holifield JS. Vascular endothelial growth factor and basic fibroblast growth factor differentially modulate early postnatal coronary angiogenesis. Circulation research. 2001;88(11):1135–41. Epub 2001/06/09. . 11397779 10.1161/hh1101.091191

[28] Morabito CJ, Dettman RW, Kattan J, Collier JM, Bristow J. Positive and negative regulation of epicardial-mesenchymal transformation during avian heart development. Developmental biology. 2001;234(1):204–15. 10.1006/dbio.2001.0254 . 11356030

[29] Craig EA, Parker P, Austin AF, Barnett JV, Camenisch TD. Involvement of the MEKK1 signaling pathway in the regulation of epicardial cell behavior by hyaluronan. Cell Signal. 2010;22(6):968–76. Epub 2010/02/18. 10.1016/j.cellsig.2010.02.004 20159036 PMC2846756

[30] Craig EA, Austin AF, Vaillancourt RR, Barnett JV, Camenisch TD. TGFbeta2-mediated production of hyaluronan is important for the induction of epicardial cell differentiation and invasion. Exp Cell Res. 2010;316(20):3397–405. Epub 2010/07/17. 10.1016/j.yexcr.2010.07.006 . 20633555 PMC3397912

[31] Shull MM, Ormsby I, Kier AB, Pawlowski S, Diebold RJ, Yin M, et al Targeted disruption of the mouse transforming growth factor-beta 1 gene results in multifocal inflammatory disease. Nature. 1992;359(6397):693–9. Epub 1992/10/22. 10.1038/359693a0 . 1436033 PMC3889166

[32] Sanford LP, Ormsby I, Gittenberger-de Groot AC, Sariola H, Friedman R, Boivin GP, et al TGFbeta2 knockout mice have multiple developmental defects that are non-overlapping with other TGFbeta knockout phenotypes. Development. 1997;124(13):2659–70. Epub 1997/07/01. . 9217007 10.1242/dev.124.13.2659 PMC3850286

[33] Sridurongrit S, Larsson J, Schwartz R, Ruiz-Lozano P, Kaartinen V. Signaling via the Tgf-beta type I receptor Alk5 in heart development. Developmental biology. 2008;322(1):208–18. Epub 2008/08/23. 10.1016/j.ydbio.2008.07.038 18718461 PMC2677203

[34] Gittenberger-de Groot AC, Winter EM, Bartelings MM, Goumans MJ, DeRuiter MC, Poelmann RE. The arterial and cardiac epicardium in development, disease and repair. Differentiation; research in biological diversity. 2012;84(1):41–53. Epub 2012/06/02. 10.1016/j.diff.2012.05.002 . 22652098

[35] Yang JT, Rayburn H, Hynes RO. Cell adhesion events mediated by alpha 4 integrins are essential in placental and cardiac development. Development. 1995;121(2):549–60. Epub 1995/02/01. . 7539359 10.1242/dev.121.2.549

[36] Tevosian SG, Deconinck AE, Tanaka M, Schinke M, Litovsky SH, Izumo S, et al FOG-2, a cofactor for GATA transcription factors, is essential for heart morphogenesis and development of coronary vessels from epicardium. Cell. 2000;101(7):729–39. Epub 2000/07/13. 10.1016/S0092-8674(00)80885-5 . 10892744

[37] Moore AW, McInnes L, Kreidberg J, Hastie ND, Schedl A. YAC complementation shows a requirement for Wt1 in the development of epicardium, adrenal gland and throughout nephrogenesis. Development. 1999;126(9):1845–57. Epub 1999/04/02. . 10101119 10.1242/dev.126.9.1845

[38] Townsend TA, Robinson JY, How T, DeLaughter DM, Blobe GC, Barnett JV. Endocardial cell epithelial-mesenchymal transformation requires Type III TGFbeta receptor interaction with GIPC. Cellular signalling. 2011;24(1):247–56. Epub 2011/09/29. 10.1016/j.cellsig.2011.09.006 21945156 PMC3208316

[39] Austin AF, Compton LA, Love JD, Brown CB, Barnett JV. Primary and immortalized mouse epicardial cells undergo differentiation in response to TGFbeta. Developmental dynamics: an official publication of the American Association of Anatomists. 2008;237(2):366–76. Epub 2008/01/24. 10.1002/dvdy.21421 . 18213583

[40] Christodoulou DC, Gorham JM, Herman DS, Seidman JG. Construction of normalized RNA-seq libraries for next-generation sequencing using the crab duplex-specific nuclease. Curr Protoc Mol Biol. Chapter 4:Unit4 12. Epub 2011/04/08. 10.1002/0471142727.mb0412s94 21472699 PMC3152986

[41] Trapnell C, Pachter L, Salzberg SL. TopHat: discovering splice junctions with RNA-Seq. Bioinformatics. 2009;25(9):1105–11. 10.1093/bioinformatics/btp120 19289445 PMC2672628

[42] Christodoulou DC, Gorham JM, Herman DS, Seidman JG. Construction of normalized RNA-seq libraries for next-generation sequencing using the crab duplex-specific nuclease. Current protocols in molecular biology / edited by Frederick M Ausubel [et al]. 2011;Chapter 4:Unit4 12. Epub 2011/04/08. 10.1002/0471142727.mb0412s94 21472699 PMC3152986

[43] Craig EA, Parker P, Camenisch TD. Size-dependent regulation of Snail2 by hyaluronan: its role in cellular invasion. Glycobiology. 2009;19(8):890–8. Epub 2009/05/20. 10.1093/glycob/cwp064 19451547 PMC2704900

[44] Craig EA, Parker P, Austin AF, Barnett JV, Camenisch TD. Involvement of the MEKK1 signaling pathway in the regulation of epicardial cell behavior by hyaluronan. Cell Signal. 2010;22(6):968–76. Epub 2010/02/18. 10.1016/j.cellsig.2010.02.004 20159036 PMC2846756

[45] Moore AW, Schedl A, McInnes L, Doyle M, Hecksher-Sorensen J, Hastie ND. YAC transgenic analysis reveals Wilms' tumour 1 gene activity in the proliferating coelomic epithelium, developing diaphragm and limb. Mech Dev. 1998;79(1–2):169–84. Epub 1999/06/01. . 10349631 10.1016/s0925-4773(98)00188-9

[46] Kraus F, Haenig B, Kispert A. Cloning and expression analysis of the mouse T-box gene Tbx20. Mechanisms of Development. 2001;100(1):87–91. 10.1016/S0925-4773(00)00499-8 . 11118890

[47] Lampugnani MG, Resnati M, Raiteri M, Pigott R, Pisacane A, Houen G, et al A Novel Endothelial-Specific Membrane-Protein Is a Marker of Cell Cell Contacts. Journal of Cell Biology. 1992;118(6):1511–22. 10.1083/jcb.118.6.1511 . 1522121 PMC2289607

[48] Newman PJ, Berndt MC, Gorski J, White GC, Lyman S, Paddock C, et al Pecam-1 (Cd31) Cloning and Relation to Adhesion Molecules of the Immunoglobulin Gene Superfamily. Science. 1990;247(4947):1219–22. 10.1126/science.1690453 . 1690453

[49] Partanen J, Armstrong E, Makela TP, Korhonen J, Sandberg M, Renkonen R, et al A Novel Endothelial-Cell Surface-Receptor Tyrosine Kinase with Extracellular Epidermal Growth-Factor Homology Domains. Molecular and Cellular Biology. 1992;12(4):1698–707. . 1312667 10.1128/mcb.12.4.1698-1707.1992 PMC369613

[50] Saggin L, Gorza L, Ausoni S, Schiaffino S. Troponin-I Switching in the Developing Heart. Journal of Biological Chemistry. 1989;264(27):16299–302. . 2777792

[51] Wang Q, Reiter RS, Huang QQ, Jin JP, Lin JJC. Comparative studies on the expression patterns of three troponin T genes during mouse development. Anatomical Record. 2001;263(1):72–84. 10.1002/Ar.1078 . 11331973

[52] Saggin L, Ausoni S, Gorza L, Sartore S, Schiaffino S. Troponin-T Switching in the Developing Rat-Heart. Journal of Biological Chemistry. 1988;263(34):18488–92. . 2973462

[53] Rensen SSM, Doevendans PAFM, van Eys GJJM. Regulation and characteristics of vascular smooth muscle cell phenotypic diversity. Netherlands Heart Journal. 2007;15(3):100–8. . 17612668 10.1007/BF03085963 PMC1847757

[54] Dennis G Jr, Sherman BT, Hosack DA, Yang J, Gao W, Lane HC, et al DAVID: Database for Annotation, Visualization, and Integrated Discovery. Genome Biol. 2003;4(5):P3 Epub 2003/05/08. . 12734009 PMC193660

[55] del Monte G, Casanova JC, Guadix JA, MacGrogan D, Burch JB, Perez-Pomares JM, et al Differential Notch signaling in the epicardium is required for cardiac inflow development and coronary vessel morphogenesis. Circulation research. 2007;108(7):824–36. Epub 2011/02/12. 10.1161/CIRCRESAHA.110.229062 . 21311046

[56] Monzani E, Bazzotti R, Perego C, La Porta CA. AQP1 is not only a water channel: it contributes to cell migration through Lin7/beta-catenin. PLoS One. 2009;4(7):e6167 Epub 2009/07/09. 10.1371/journal.pone.0006167 19584911 PMC2701997

[57] Troyanovsky B, Levchenko T, Mansson G, Matvijenko O, Holmgren L. Angiomotin: an angiostatin binding protein that regulates endothelial cell migration and tube formation. J Cell Biol. 2001;152(6):1247–54. Epub 2001/03/21. 11257124 10.1083/jcb.152.6.1247 PMC2199208

[58] Sato T, Gotoh M, Kiyohara K, Akashima T, Iwasaki H, Kameyama A, et al Differential roles of two N-acetylgalactosaminyltransferases, CSGalNAcT-1, and a novel enzyme, CSGalNAcT-2. Initiation and elongation in synthesis of chondroitin sulfate. The Journal of biological chemistry. 2003;278(5):3063–71. Epub 2002/11/26. 10.1074/jbc.M208886200 . 12446672

[59] Ruffell B, Poon GF, Lee SS, Brown KL, Tjew SL, Cooper J, et al Differential use of chondroitin sulfate to regulate hyaluronan binding by receptor CD44 in Inflammatory and Interleukin 4-activated Macrophages. The Journal of biological chemistry. 2011;286(22):19179–90. Epub 2011/04/08. 10.1074/jbc.M110.200790 21471214 PMC3103297

[60] Rosati B, Grau F, McKinnon D. Regional variation in mRNA transcript abundance within the ventricular wall. J Mol Cell Cardiol. 2006;40(2):295–302. Epub 2006/01/18. 10.1016/j.yjmcc.2005.11.002 . 16412459

[61] Bochmann L, Sarathchandra P, Mori F, Lara-Pezzi E, Lazzaro D, Rosenthal N. Revealing new mouse epicardial cell markers through transcriptomics. PLoS One. 2010;5(6):e11429 Epub 2010/07/03. 10.1371/journal.pone.0011429 20596535 PMC2893200

[62] Buermans HP, van Wijk B, Hulsker MA, Smit NC, den Dunnen JT, van Ommen GB, et al Comprehensive gene-expression survey identifies wif1 as a modulator of cardiomyocyte differentiation. PLoS One. 2010;5(12):e15504 Epub 2010/12/24. 10.1371/journal.pone.0015504 21179454 PMC3001492

[63] Smith CL, Baek ST, Sung CY, Tallquist MD. Epicardial-derived cell epithelial-to-mesenchymal transition and fate specification require PDGF receptor signaling. Circulation research. 2011;108(12):e15–26. Epub 2011/04/23. 10.1161/CIRCRESAHA.110.235531 21512159 PMC3134964

[64] Allison P, Espiritu D, Barnett JV, Camenisch TD. Type III TGFbeta receptor and Src direct hyaluronan-mediated invasive cell motility. Cell Signal. 2015;27(3):453–9. 10.1016/j.cellsig.2014.11.037 . 25499979 PMC5604324

[65] Kalman F, Viragh S, Modis L. Cell surface glycoconjugates and the extracellular matrix of the developing mouse embryo epicardium. Anat Embryol (Berl). 1995;191(5):451–64. Epub 1995/05/01. . 7625614 10.1007/BF00304430

[66] Tjew SL, Brown KL, Kannagi R, Johnson P. Expression of N-acetylglucosamine 6-O-sulfotransferases (GlcNAc6STs)-1 and -4 in human monocytes: GlcNAc6ST-1 is implicated in the generation of the 6-sulfo N-acetyllactosamine/Lewis x epitope on CD44 and is induced by TNF-alpha. Glycobiology. 2005;15(7):7C–13C. Epub 2005/02/25. 10.1093/glycob/cwi050 . 15728736

[67] Ruffell B, Johnson P. Chondroitin sulfate addition to CD44H negatively regulates hyaluronan binding. Biochem Biophys Res Commun. 2005;334(2):306–12. Epub 2005/07/09. 10.1016/j.bbrc.2005.06.108 . 16002044

[68] Tidball JG. Identification and distribution of a novel, collagen-binding protein in the developing subepicardium and endomysium. The Journal of biological chemistry. 1992;267(29):21211–9. Epub 1992/10/15. . 1328225

[69] Zanin MK, Bundy J, Ernst H, Wessels A, Conway SJ, Hoffman S. Distinct spatial and temporal distributions of aggrecan and versican in the embryonic chick heart. Anat Rec. 1999;256(4):366–80. Epub 1999/12/10. 10.1002/(SICI)1097-0185 . 10589023

[70] Ricciardelli C, Sakko AJ, Ween MP, Russell DL, Horsfall DJ. The biological role and regulation of versican levels in cancer. Cancer Metastasis Rev. 2009;28(1–2):233–45. Epub 2009/01/23. 10.1007/s10555-009-9182-y . 19160015

[71] Mjaatvedt CH, Yamamura H, Capehart AA, Turner D, Markwald RR. The Cspg2 gene, disrupted in the hdf mutant, is required for right cardiac chamber and endocardial cushion formation. Developmental biology. 1998;202(1):56–66. Epub 1998/10/06. 10.1006/dbio.1998.9001 . 9758703

[72] Kern CB, Twal WO, Mjaatvedt CH, Fairey SE, Toole BP, Iruela-Arispe ML, et al Proteolytic cleavage of versican during cardiac cushion morphogenesis. Developmental dynamics: an official publication of the American Association of Anatomists. 2006;235(8):2238–47. Epub 2006/05/13. 10.1002/dvdy.20838 16691565 PMC1828280

[73] Schlueter J, Manner J, Brand T. BMP is an important regulator of proepicardial identity in the chick embryo. Developmental biology. 2006;295(2):546–58. Epub 2006/05/09. 10.1016/j.ydbio.2006.03.036 . 16677627

[74] Ishii Y, Garriock RJ, Navetta AM, Coughlin LE, Mikawa T. BMP signals promote proepicardial protrusion necessary for recruitment of coronary vessel and epicardial progenitors to the heart. Dev Cell. 2010;19(2):307–16. Epub 2010/08/17. 10.1016/j.devcel.2010.07.017 20708592 PMC2925255

[75] Ernkvist M, Luna Persson N, Audebert S, Lecine P, Sinha I, Liu M, et al The Amot/Patj/Syx signaling complex spatially controls RhoA GTPase activity in migrating endothelial cells. Blood. 2009;113(1):244–53. Epub 2008/10/01. 10.1182/blood-2008-04-153874 18824598 PMC2614636

[76] Zheng Y, Vertuani S, Nystrom S, Audebert S, Meijer I, Tegnebratt T, et al Angiomotin-like protein 1 controls endothelial polarity and junction stability during sprouting angiogenesis. Circulation research. 2009;105(3):260–70. Epub 2009/07/11. 10.1161/CIRCRESAHA.109.195156 . 19590046

[77] Masuda M, Maruyama T, Ohta T, Ito A, Hayashi T, Tsukasaki K, et al CADM1 interacts with Tiam1 and promotes invasive phenotype of human T-cell leukemia virus type I-transformed cells and adult T-cell leukemia cells. The Journal of biological chemistry. 2010;285(20):15511–22. Epub 2010/03/11. 10.1074/jbc.M109.076653 20215110 PMC2865322

[78] Attar MA, Salem JC, Pursel HS, Santy LC. CNK3 and IPCEF1 produce a single protein that is required for HGF dependent Arf6 activation and migration. Exp Cell Res. 2012;318(3):228–37. Epub 2011/11/17. 10.1016/j.yexcr.2011.10.018 . 22085542

[79] Perego C, Vanoni C, Massari S, Raimondi A, Pola S, Cattaneo MG, et al Invasive behaviour of glioblastoma cell lines is associated with altered organisation of the cadherin-catenin adhesion system. J Cell Sci. 2002;115(Pt 16):3331–40. Epub 2002/07/26. . 12140264 10.1242/jcs.115.16.3331

[80] Zhou B, Honor LB, He H, Ma Q, Oh JH, Butterfield C, et al Adult mouse epicardium modulates myocardial injury by secreting paracrine factors. The Journal of clinical investigation. 2011;121(5):1894–904. Epub 2011/04/21. 10.1172/JCI45529 21505261 PMC3083761

[81] You HJ, How T, Blobe GC. The type III transforming growth factor-beta receptor negatively regulates nuclear factor kappa B signaling through its interaction with beta-arrestin2. Carcinogenesis. 2009;30(8):1281–7. Epub 2009/03/28. 10.1093/carcin/bgp071 19325136 PMC2718069

[82] Lim S, Bae E, Kim HS, Kim TA, Byun K, Kim B, et al TRAF6 mediates IL-1beta/LPS-induced suppression of TGF-beta signaling through its interaction with the type III TGF-beta receptor. PLoS One. 2012;7(3):e32705 Epub 2012/03/20. 10.1371/journal.pone.0032705PONE-D-11-19939 22427868 PMC3299683

[83] Verstrepen L, Bekaert T, Chau TL, Tavernier J, Chariot A, Beyaert R. TLR-4, IL-1R and TNF-R signaling to NF-kappaB: variations on a common theme. Cell Mol Life Sci. 2008;65(19):2964–78. Epub 2008/06/07. 10.1007/s00018-008-8064-8 . 18535784 PMC11131687

[84] Tauseef M, Knezevic N, Chava KR, Smith M, Sukriti S, Gianaris N, et al TLR4 activation of TRPC6-dependent calcium signaling mediates endotoxin-induced lung vascular permeability and inflammation. J Exp Med. 2012;209(11):1953–68. Epub 2012/10/10. 10.1084/jem.20111355 23045603 PMC3478927

[85] Kawagoe T, Sato S, Matsushita K, Kato H, Matsui K, Kumagai Y, et al Sequential control of Toll-like receptor-dependent responses by IRAK1 and IRAK2. Nat Immunol. 2008;9(6):684–91. Epub 2008/04/29. 10.1038/ni.1606 . 18438411

[86] Medvedev AE, Lentschat A, Wahl LM, Golenbock DT, Vogel SN. Dysregulation of LPS-induced Toll-like receptor 4-MyD88 complex formation and IL-1 receptor-associated kinase 1 activation in endotoxin-tolerant cells. J Immunol. 2002;169(9):5209–16. Epub 2002/10/23. . 12391239 10.4049/jimmunol.169.9.5209

[87] Cole WC, Welsh DG. Role of myosin light chain kinase and myosin light chain phosphatase in the resistance arterial myogenic response to intravascular pressure. Arch Biochem Biophys. 2011;510(2):160–73. Epub 2011/03/12. 10.1016/j.abb.2011.02.024 . 21392499

[88] Kamm KE, Stull JT. Dedicated myosin light chain kinases with diverse cellular functions. The Journal of biological chemistry. 2001;276(7):4527–30. Epub 2000/11/30. 10.1074/jbc.R000028200 . 11096123

[89] Wang L, Hauser ER, Shah SH, Pericak-Vance MA, Haynes C, Crosslin D, et al Peakwide mapping on chromosome 3q13 identifies the kalirin gene as a novel candidate gene for coronary artery disease. Am J Hum Genet. 2007;80(4):650–63. Epub 2007/03/16. 10.1086/512981 17357071 PMC1852708

[90] Criswell TL, Arteaga CL. Modulation of NFkappaB activity and E-cadherin by the type III transforming growth factor beta receptor regulates cell growth and motility. The Journal of biological chemistry. 2007;282(44):32491–500. Epub 2007/09/08. 10.1074/jbc.M704434200 . 17823118

[91] Criswell TL, Dumont N, Barnett JV, Arteaga CL. Knockdown of the transforming growth factor-beta type III receptor impairs motility and invasion of metastatic cancer cells. Cancer research. 2008;68(18):7304–12. Epub 2008/09/17. 10.1158/0008-5472.CAN-07-6777 . 18794117

[92] Clark CR, Robinson JY, Sanchez NS, Townsend TA, Arrieta JA, Merryman WD, et al Common pathways regulate Type III TGFbeta receptor-dependent cell invasion in epicardial and endocardial cells. Cellular signalling. 2016;28(6):688–98. Epub 2016/03/13. 10.1016/j.cellsig.2016.03.004 26970186 PMC4827451

[93] Brown CB, Boyer A, Runyan RB, Barnett JV. Requirement of the Type III TGF receptor for endocardial cell transformation in the heart. Science 1999;283(5410):2080–2082. 10.1126/science.283.5410.2080 . 10092230

[94] Audic S, Claverie JM. The significance of digital gene expression profiles. Genome Res. 1997;7(10):986–95. Epub 1997/10/23. . 9331369 10.1101/gr.7.10.986

