# Supplementary figures and images for: Transcriptional Profiling of Cultured, Embryonic Epicardial Cells Identifies Novel Genes and Signaling Pathways Regulated by TGFβR3 In Vitro
Source: PLoS One. 2016 Aug 9;11(8):e0159710. doi: 10.1371/journal.pone.0159710 (PMC4978490; doi:10.1371/journal.pone.0159710)

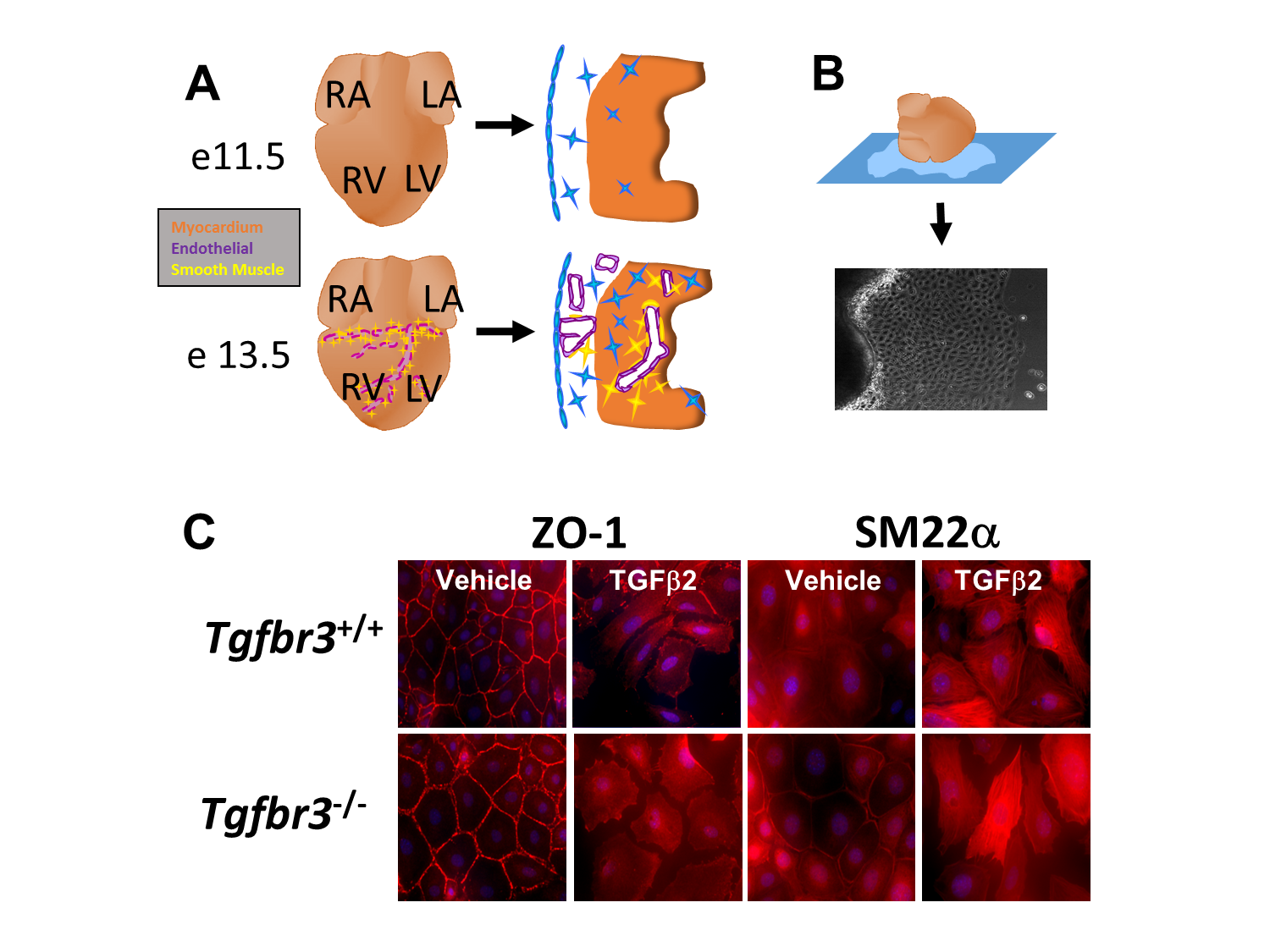

Supplement: S1 Fig — The reads for the two biological replicates (n = 1, n = 2) for each group (VEH, TGFβ1, TGFβ2, BMP2) in Tgfbr3+/+ (A-D) or Tgfbr3-/- (E-H) were plotted against each other. There was a high degree of agreement in Tgfbr3+/+ (A-D) (R>0.87) or Tgfbr3-/- (E-H) (R>0.89) datasets. These comparisons support a high degree of agreement between biological replicates. (TIF) [file pone.0159710.s001.tif]

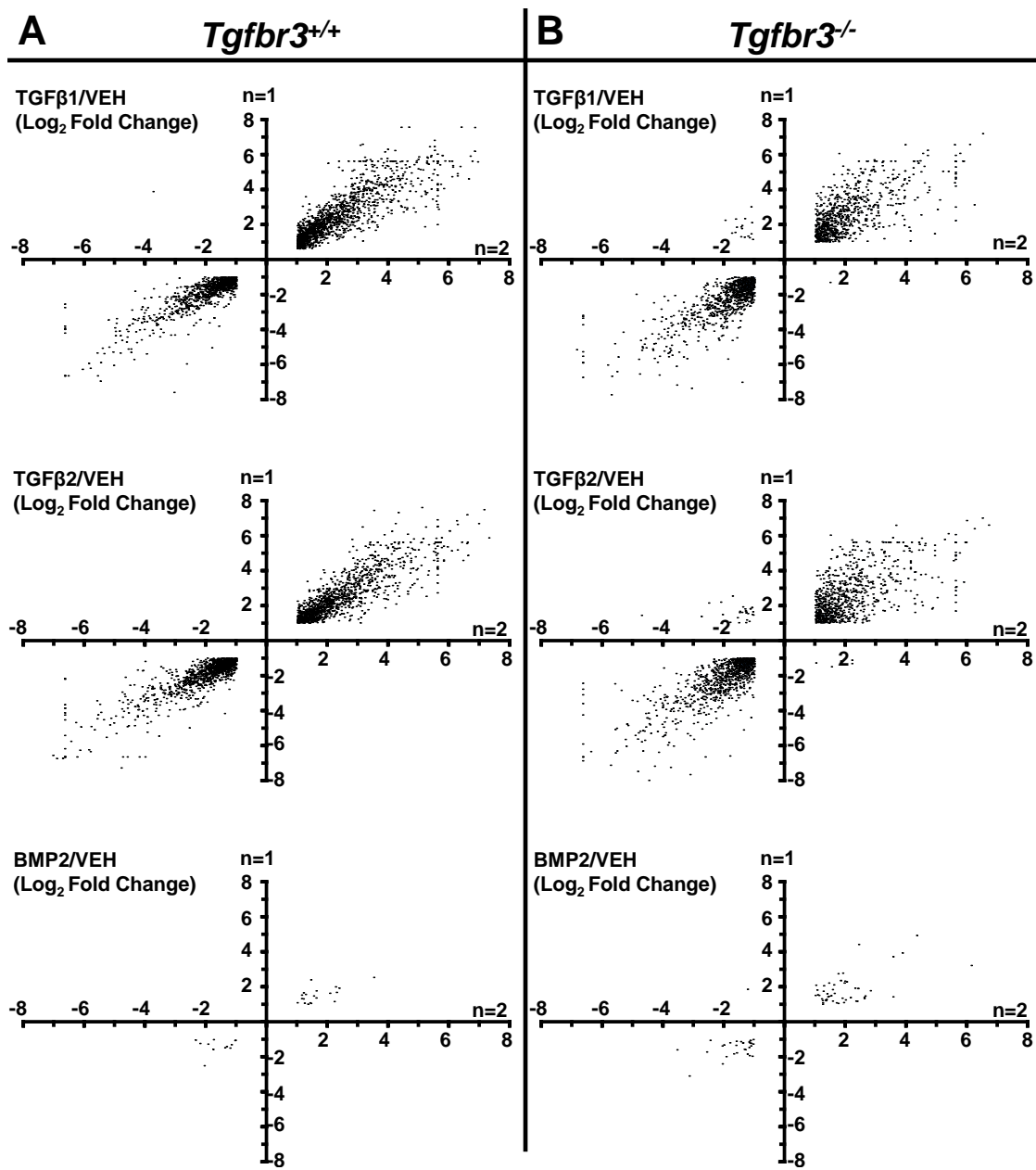

Supplement: S2 Fig — Plots mapping the fold (log base 2) difference >2-fold in expression between VEH and ligand incubated groups in Tgfbr3+/+ (A) or Tgfbr3-/- (B) in biological replicates (X-axis: n = 1, Y-axis: n = 2) shown. Genes that have agreement, defined as having >2-fold (p<0.001) increased or decreased expression in a specific comparison in both replicates, are mapped to quadrants I (upper right) or III (lower left) of a plot. Genes that show disagreement, defined as having >2-fold (p<0.001) increased expression in a tissue in one replicate and decreased in another (or vis versa), are mapped to quadrants II (upper left) or IV (lower right). There was a high degree of agreement in Tgfbr3+/+ (A) (R>0.85) or Tgfbr3-/- (B) (R>0.89) datasets across all comparisons [94]. Variability between biological replicates was determined. (PDF) [file pone.0159710.s002.pdf]

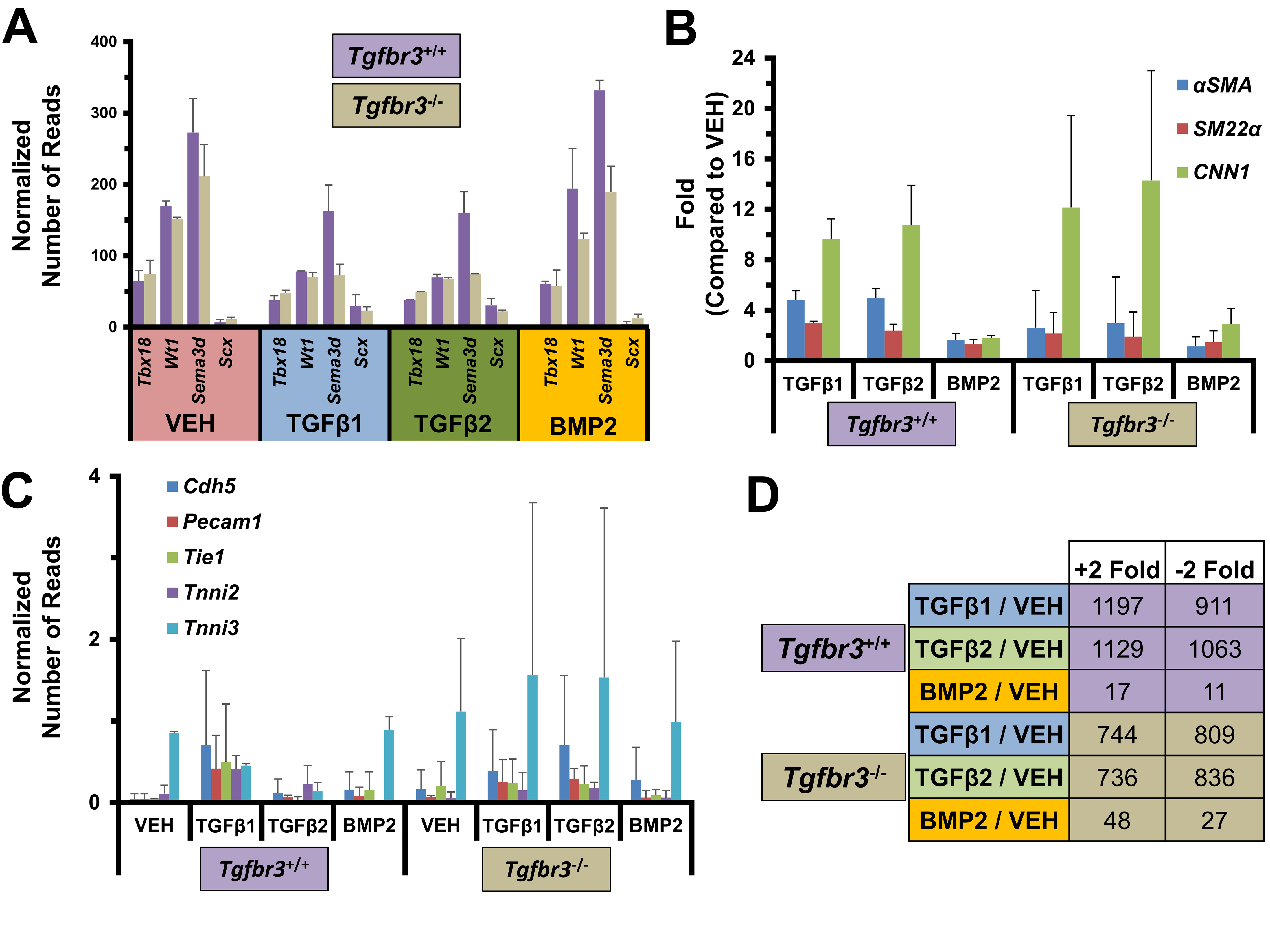

Supplement: S3 Fig — Differential gene expression between Tgfbr3+/+ and Tgfbr3-/- epicardial cells observed in RNA-seq data was evaluated using qRT-PCR analysis (n = 3). Expression was normalized to the constitutive expression level of GAPDH RNA and the ratio of transcriptional abundance found in Tgfbr+/+ to Tgfbr-/- is depicted. (TIF) [file pone.0159710.s003.tif]
