# Supplementary material for: Transcriptional Profiling of Cultured, Embryonic Epicardial Cells Identifies Novel Genes and Signaling Pathways Regulated by TGFβR3 In Vitro
Source: PLoS One. 2016 Aug 9;11(8):e0159710. doi: 10.1371/journal.pone.0159710 (PMC4978490; doi:10.1371/journal.pone.0159710)

A

TGFβ1	
**Adhesion**	
GO:0007155~cell adhesion<br>GO:0022610~biological adhesion	*Itgbl1, Klra4, Fat2, Pkp1*
**Bone and Cartilage Development**	
GO:0048705~skeletal system morphogenesis<br>GO:0048704~emb. skeletal system morphogenesis	*Gas1, Dlx2, Tbx15, Tbx4, Wnt7a, Hoxa5*
**Blood Vessel and Vasculature Development**	
GO:0001944~vasculature development<br>GO:0048514~blood vessel morphogenesis	*Fgf2, Fgf10, Scg2, Sema5a*
**Proliferation**	
GO:0008284~positive regulation of cell proliferation	*Cd80, Gli1, Ereg, Sp6, Clu*
**Lung Development**	
GO:0030324~lung development<br>GO:0060541~respiratory system development	Tcf21, Id1, Hsd11b1, Foxp2,,Mgp
**ECM**	
GO:0043062~extracellular structure organization	Adamts14, Adamts20
**Epithelial Development**	
GO:0060429~epithelium development<br>GO:0030855~epithelial cell differentiation	*Irf6, Nkx2-3, Sfrp1, Id3*
**Brain Development**	
GO:0030902~hindbrain development<br>GO:0022037~metencephalon development	*Phox2a, Smad9, Lhx5, Cacna1a*

B

VEH	
**Adhesion**	
GO:0007155~cell adhesion<br>GO:0022610~biological adhesion<br>GO:0016337~cell-cell adhesion	*Cadm1, Alcam1, CDH26, CDH13*
**Bone and Cartilage Development**	
GO:0048705~skeletal system morphogenesis<br>GO:0001501~skeletal system development<br>GO:0051216~cartilage development<br>GO:0048729~tissue morphogenesis<br>GO:0048704~emb.keletal system morphogenesis<br>GO:0048706~emb. skeletal system development	*Bmpr1b, Hoxb2, Alx4, Prrx2, Wnt9a, Col11a1, Mgp, P2rx7*
**Blood Vessel and Vasculature Development**	
GO:0001568~blood vessel development<br>GO:0001944~vasculature development<br>GO:0048514~blood vessel morphogenesis<br>GO:0001569~patterning of blood vessels	Fgf8, Fgf9, Fgf10, Fgf18, Sema3c
**Proliferation**	
GO:0008284~positive regulation of cell proliferation<br>GO:0042127~regulation of cell proliferation<br>GO:0008283~cell proliferation	*Lif, Ccnd2, Esr1, Nog, Hlx, Irf6,*
**ECM**	
GO:0043062~extracellular structure organization<br>GO:0030198~extracellular matrix organization	*Col18a1, Tnc, Eln, Col5a3, Col4a3, Fbln5*
**Patterning**	
GO:0001763~morphogenesis of a branching structure<br>GO:0007389~pattern specification process<br>GO:0003002~regionalization	Hoxb3, Hoxb4, Foxc2, Flt1, Wnt3, Tbx20

C TGFβ1

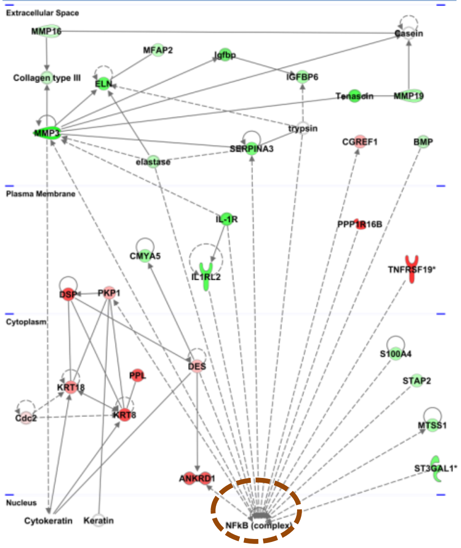

D VEH

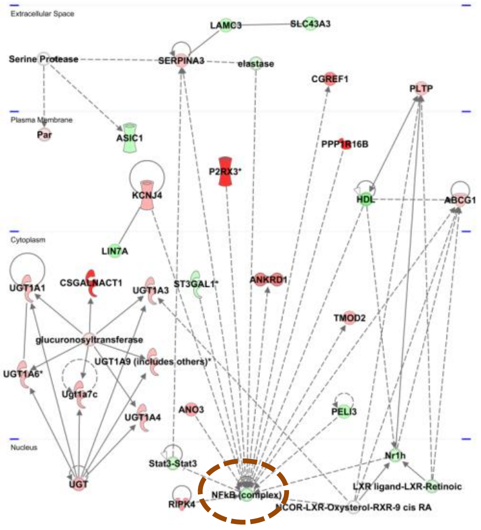

Supplement: S4 Fig — Genes >2-fold (p<0.001) differentially expressed between Tgfbr3+/+ and Tgfbr3-/- epicardial cells incubated with either TGFβ1 (A) or VEH (B) were subjected (A) to gene ontology analysis (using DAVID software, p<0.0001). (C-D) NF-ĸB signaling (orange circle) is a central node in representative networks generated by gene regulatory network analysis (using Ingenuity Pathway Analysis software). Green- expressed higher inTgfbr3+/+, Red- expressed higher in Tgfbr3-/-. (PDF) [file pone.0159710.s004.pdf]
