# Supplementary material for: Transcriptional Profiling of Cultured, Embryonic Epicardial Cells Identifies Novel Genes and Signaling Pathways Regulated by TGFβR3 In Vitro
Source: PLoS One. 2016 Aug 9;11(8):e0159710. doi: 10.1371/journal.pone.0159710 (PMC4978490; doi:10.1371/journal.pone.0159710)
Supplement: S5 Fig — Genes identified as being >2-fold differentially regulated between Tgfbr3+/+ or Tgfbr3-/- epicardial cells incubated with (A) VEH, (B) BMP2, (C) TGFβ1, or (D) TGFβ2. Solid lines denote a direct interaction while dotted lines denote indirect interaction between proteins. Green- higher expression in Tgfbr3+/+. Red- higher expression in Tgfbr3-/-. (PDF) [file pone.0159710.s005.pdf]

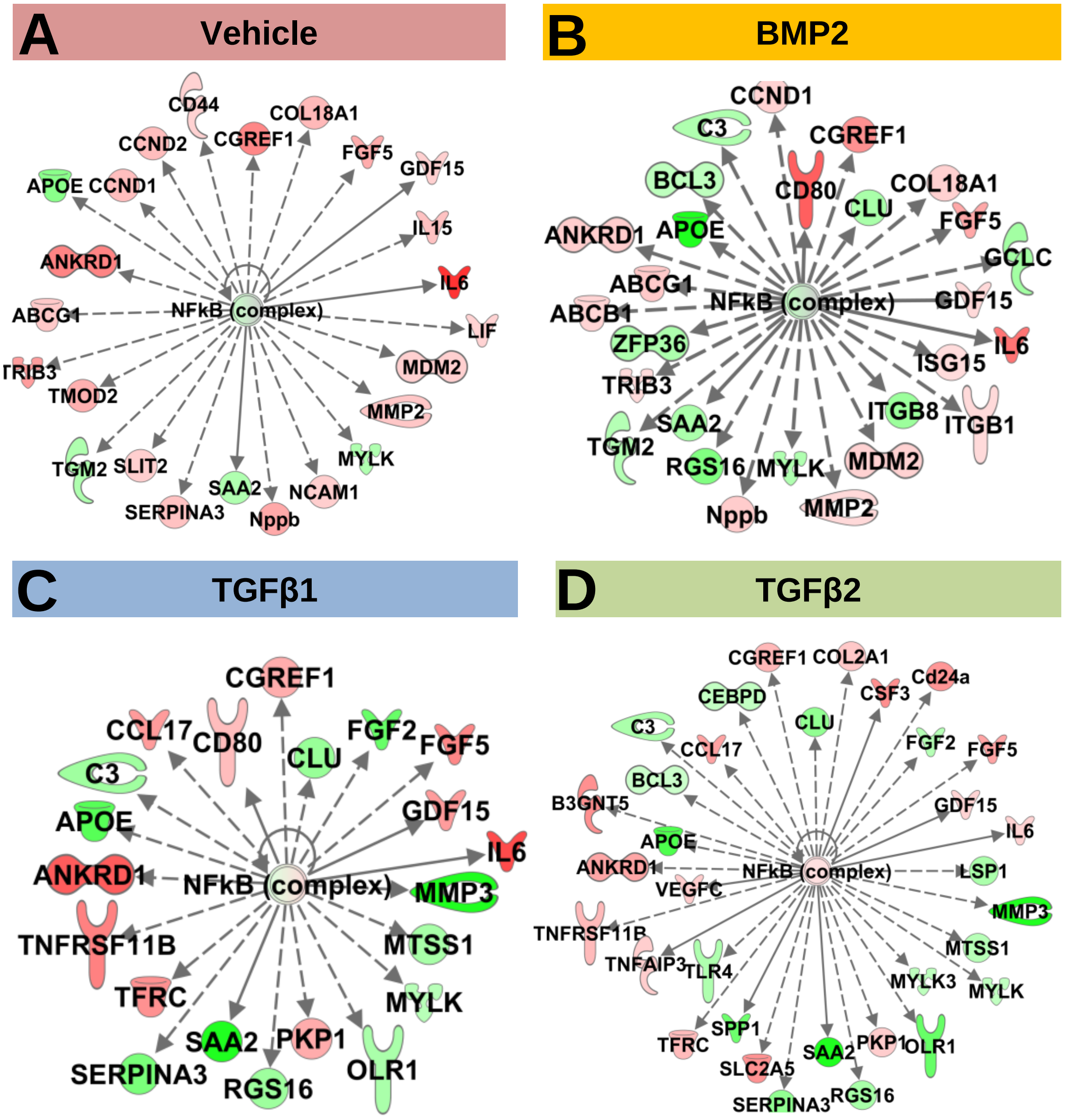
A
Vehicle
CD44
COL18A1
CCND2
CGREF1
FGF5
GDF15
APOE
CCND1
IL15
ANKRD1
IL6
ABCG1
NFkB (complex)
LIF
TRIB3
MDM2
TMOD2
MMP2
MYLK
TGM2
SLIT2
SAA2
NCAM1
SERPINA3
Nppb
B
BMP2
CCND1
C3
CGREF1
BCL3
CD80
COL18A1
CLU
ANKRD1
APOE
FGF5
GCLC
ABCG1
ABCB1
NFkB (complex)
GDF15
ZFP36
IL6
ISG15
TRIB3
ITGB8
ITGB1
SAA2
TGM2
RGS16
MYLK
MDM2
Nppb
MMP2
C
TGFβ1
CGREF1
CCL17
CD80
FGF2
FGF5
CLU
C3
GDF15
APOE
IL6
ANKRD1
NFkB (complex)
MMP3
TNFRSF11B
MTSS1
TFRC
MYLK
SAA2
PKP1
SERPINA3
RGS16
OLR1
D
TGFβ2
CGREF1
COL2A1
Cd24a
CEBPD
CSF3
C3
CLU
CCL17
FGF2
FGF5
BCL3
B3GNT5
GDF15
APOE
IL6
ANKRD1
NFkB (complex)
LSP1
VEGFC
MMP3
TNFRSF11B
MTSS1
TNFAIP3
TLR4
MYLK3
MYLK
SPP1
TFRC
SAA2
PKP1
OLR1
SLC2A5
SERPINA3
RGS16
